# Broadly neutralizing humanized SARS-CoV-2 antibody binds to a conserved epitope on Spike and provides antiviral protection through inhalation-based delivery in non-human primates

**DOI:** 10.1371/journal.ppat.1011532

**Published:** 2023-08-02

**Authors:** Paule Hermet, Benoît Delache, Cecile Herate, Esther Wolf, Gaily Kivi, Erkki Juronen, Karl Mumm, Eva Žusinaite, Denis Kainov, Eve Sankovski, Kai Virumäe, Anu Planken, Andres Merits, Jessica E. Besaw, Ai Woon Yee, Takefumi Morizumi, Kyumhyuk Kim, Anling Kuo, Asma Berriche, Nathalie Dereuddre-Bosquet, Quentin Sconosciuti, Thibaut Naninck, Francis Relouzat, Mariangela Cavarelli, Mart Ustav, Derek Wilson, Oliver P. Ernst, Andres Männik, Roger LeGrand, Mart Ustav

**Affiliations:** 1 Icosagen Cell Factory OÜ; Tartu, Estonia; 2 Université Paris-Saclay, Inserm, CEA, Center for Immunology of Viral, Auto-immune, Hematological and Bacterial Diseases (IMVA-HB/IDMIT); Fontenay-aux-Roses, France; 3 York University; Toronto, Canada; 4 University of Tartu; Tartu, Estonia; 5 NTNU; Trondheim, Norway; 6 Department of Biochemistry, University of Toronto; Toronto, Canada; 7 Department of Molecular Genetics, University of Toronto; Toronto, Canada; Leiden University Medical Center: Leids Universitair Medisch Centrum, NETHERLANDS

## Abstract

The COVID-19 pandemic represents a global challenge that has impacted and is expected to continue to impact the lives and health of people across the world for the foreseeable future. The rollout of vaccines has provided highly anticipated relief, but effective therapeutics are required to further reduce the risk and severity of infections. Monoclonal antibodies have been shown to be effective as therapeutics for SARS-CoV-2, but as new variants of concern (VoC) continue to emerge, their utility and use have waned due to limited or no efficacy against these variants. Furthermore, cumbersome systemic administration limits easy and broad access to such drugs. As well, concentrations of systemically administered antibodies in the mucosal epithelium, a primary site of initial infection, are dependent on neonatal Fc receptor mediated transport and require high drug concentrations. To reduce the viral load more effectively in the lung, we developed an inhalable formulation of a SARS-CoV-2 neutralizing antibody binding to a conserved epitope on the Spike protein, ensuring pan-neutralizing properties. Administration of this antibody via a vibrating mesh nebulization device retained antibody integrity and resulted in effective distribution of the antibody in the upper and lower respiratory tract of non-human primates (NHP). In comparison with intravenous administration, significantly higher antibody concentrations can be obtained in the lung, resulting in highly effective reduction in viral load post SARS-CoV-2 challenge. This approach may reduce the barriers of access and uptake of antibody therapeutics in real-world clinical settings and provide a more effective blueprint for targeting existing and potentially emerging respiratory tract viruses.

## Author summary

COVID-19, caused by the SARS-CoV-2 virus, has had a global impact since the beginning of 2020. Although vaccines were developed and accepted under emergency procedures in under a year–certain populations still remain vulnerable for the viral infection. Monoclonal antibodies could be very potent against viral infections and respiratory diseases, but SARS-CoV-2 has presented many challenges in that field. One being the speed of emerging mutations, which has prevented many developed antibodies from working against later variants, and the other is the route of administration. Here, we developed two monoclonal antibodies of which one lost its virus neutralizing activity due to the mutations in Spike protein. As the second antibody maintained its effectiveness despite the mutational dynamics, we tested it in a viral challenge with non-human primates. We show that nebulization of therapeutic antibody provides significantly higher antibody concentrations in the lungs and airways, where most of the viral replication takes place. Therefore, nebulization could provide a more accessible and effective way of managing respiratory viruses, minimizing potential side effects and cost.

## Introduction

The ongoing COVID-19 pandemic, which started in Wuhan, China in 2019, has spread globally (www.who.int) and still represents a threat to public health. In addition to serious implications for human health, the viral pandemic has caused an excessive economic burden. Although several vaccines have been approved, there is still a need for prophylactic and therapeutic approaches, in particular, for patients who may not respond appropriately to vaccines, such as immunocompromised individuals. The continuous rise of new more infectious mutational variants of SARS-CoV-2 that have higher resistance to monoclonal antibody (mAb)-based therapeutics and vaccines are further fueling the urgency of rapid measures to limit the spread of SARS-CoV-2 [1–3]. Current evidence indicates that the emergency-use approval of recombinant mAbs that bind to the viral trimeric S-protein and prevent binding to the host receptor angiotensin-converting enzyme 2 (ACE2) have had a preliminary clinical impact in limiting the development of severe COVID-19 when administered during the early onset of infection [4,5]. However, use of mAbs as therapeutic agents represent important limitations including intravenous (IV) administration, the need to obtain high-level plasma concentrations, high cost and as well as antigenic drift and potential for neutralization escape mutations. The latter has shown a great challenge in the few years in development of neutralizing SARS-CoV-2 antibodies.

The SARS-CoV-2 virus displays 25 to 100 molecules of Spike (S) proteins (Spike trimer) on the viral membrane. Spikes interact with the host cell-surface proteins ACE2, neuropilin-1, and serine protease TMPRSS2 to facilitate viral entry into the cells [6–9]. Numerous antibodies have been reported that bind to various epitopes on the Spike trimer, blocking viral entry into the cells [10–18]. Most antibodies bind to the receptor-binding domain (RBD) of the S-protein, which can be found either in an upward ACE2 accessible or downward ACE2 inaccessible conformation [19], and block the interaction with ACE2. As for other RNA genome viruses, the SARS-CoV-2 viral genome undergoes random mutations that can alter viral fitness, and multiple variants have been reported since the emergence of the pandemic [3,20,21]. Distinct variants have appeared in certain regions before becoming widely prevalent due to better fitness. The latest highly divergent Omicron (B.1.1.529) which emerged from South-Africa at the end of 2021, has now developed multiple sub-lineages showing significant growth advantage. These variants mostly contain mutations located in the Spike protein, causing implications for the development of antibody-based therapies. Indeed, specific mutations, particularly in the RBD region, may limit the ability of antibodies to neutralize the virus, raising concerns about potential reinfection [1,22,23].

In the treatment and management of respiratory diseases caused by various pathogens, including viruses, both inhalation-based delivery and therapeutic antibodies are becoming increasingly relevant [24]. For the delivery of mAbs, the systemic route is most commonly used. The transport of IgG antibodies from the blood to the lungs is dependent on receptor-mediated transcytosis via neonatal Fc receptors (FcRn) expressed on epithelial cells of the upper airways and alveolar macrophages in humans, as well as in macaques [25,26]. However, IV administration of antibodies results in significantly lower antibody concentrations in bronchoalveolar lavage (BAL) than serum [27], whereas the inhalation-based delivery of drugs is considered to ensure an immediate high drug concentration in the target tissues, allowing the drug to act quickly, using a lower overall dose and at a lower cost. Nebulization (Neb.) as a delivery method is, thus, an attractive alternative to the common IV route, as it is non-invasive and provides local drug delivery with minimal systemic side effects, limiting drug exposure to secondary organs [28]. Moreover in the context of SARS-CoV-2, it replicates abundantly in upper airways, therefore nebulization of therapeutic antibodies is the fastest method for on-site antibody delivery [29].

Here, we report the development pipeline of highly potent SARS-CoV-2 neutralizing antibodies, obtained from both convalescent patient sera and immunized rabbits. Humanization of the isolated rabbit antibody did not alter the highly effective SARS-CoV-2 neutralization neither *in vitro* nor *in vivo* against all variant of concerns (VoCs) known to date. Pan-neutralization is most likely caused by binding to a conserved epitope on S-protein, confirmed by hydrogen-deuterium exchange mass spectrometry analysis (HDX-MS). We managed to retain antibody integrity post-nebulization and further demonstrate the potential of inhalation-based delivery of such recombinant mAbs, which could overcome many of the limitations related to IV delivery, on a wider scale.

## Results

### Antibody discovery from convalescent patients using the HybriFree platform, the *in vivo* efficacy in Syrian golden hamsters

**Fig 1:**
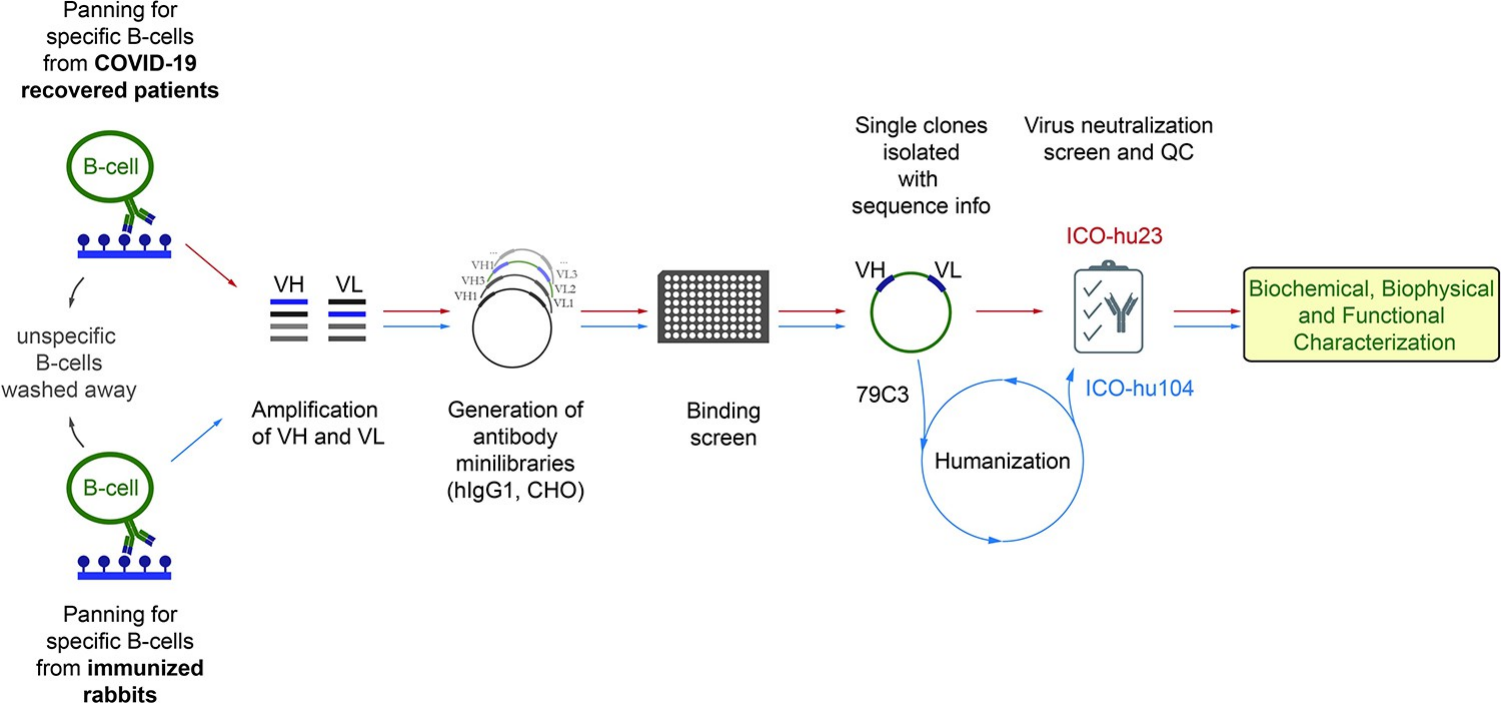
Schematic overview of the HybriFree antibody-cloning procedure and antibody validation workflow. B-cells from blood of the recovered patient or splenocytes from immunized rabbits were enriched for S-protein antigens and used to develop recombinant antibody expression libraries. Libraries with target specificity were further isolated to single clones followed by biochemical and functional characterization.

In April 2020 at the beginning of SARS-CoV-2 outbreak, we recruited 123 convalescent patients in Estonia to obtain antibodies from their B cells during convalescence. B cells from patients with the highest serum antibody virus neutralization potential were selected for cloning genes encoding for antibodies using the HybriFree technology [30], which included enrichment of the source B cell towards those cells expressing antibodies against the S1-domain or the RBD of S-protein (Fig 1). HybriFree technology enables isolation of specific, high-affinity antibodies through panning and multiple washing steps without requiring B cell culturing nor single cell manipulations. Therefore, this technology could be applicable to any species for which antibody cDNA sequences are available [30].

We screened the neutralization efficacy of obtained antibodies using VERO E6 cells in a cytopathic effect assay (S1A Fig), as well as ELISA-based binding EC50 (S1B Fig) and binding kinetics by bio-layer interferometry (BLI) were determined for the virus neutralizing antibodies. Most antibodies showed a low nanomolar apparent K_D_ against the monomeric S1 domain and a low picomolar K_D_ against the Spike trimer (S1C Fig). The antibody ICO-hu23 showed full protection effect in concentration of 16.48 pM in the CPE assay and represented the most potent SARS-CoV-2 neutralizing antibody in our panel (S1A Fig).

Syrian golden hamsters are a relevant model for SARS-CoV-2 infection, with many symptoms similar to those reported for humans–rapid breathing, weight loss, and cough [31,32]. The viral load can be detected in multiple locations in the respiratory tract, including the lungs, within the first week of viral challenge. We evaluated the efficacy of antibody ICO-hu23 at three different concentrations (n = 8)– 5 mg/kg, 0.75 mg/kg, and 0.1 mg/kg as both prophylactic and therapeutic treatments of SARS-CoV-2 infection. Intraperitoneal administration of antibody ICO-hu23 in Syrian golden hamsters showed anti-viral efficacy both 24 h before (prophylactic treatment) and after (therapeutic settings) infection with 10^5^ pfu of SARS-CoV-2 (Slovakia/SK-BMC5/2020) (S2A Fig) as assessed four days post-infection by lung viral load by RT-PCR (S2B Fig as prophylactic and S2C Fig as therapeutic).

### Antibody discovery from immunized rabbits using the HybriFree platform

As new variants of SARS-CoV-2 continued to emerge, antibody ICO-hu23 showed vulnerability to certain mutations as confirmed by pseudovirus assay in S3 Fig. Therefore, we conducted a new round of screening, using material from immunized rabbits, to find antibodies with virus-neutralizing properties for the emerging virus variants. Immunization, discovery, and the isolation of mAbs that recognize the SARS-CoV-2 Wuhan strain S1 protein were performed using HybriFree technology [30]. New-Zealand white rabbits were immunized with the RBD fragment and boosted with the S1 fragment of the Wuhan strain. We performed an ELISA-based ACE2 blocking assay to screen and select isolated antibody clones which block the binding between Spike protein of Beta variant (B.1.351) and ACE-2. Among 21 isolated rabbit antibody clones, two showed concentration-dependent neutralization and one, 79C3, was chosen for further development (S4 Fig). We humanized the antibody with the intention of using it as a therapeutic agent in humans. A chimera of the rabbit antibody 79C3 (IgG-ĸ), in which the rabbit antibody constant regions were replaced with the human antibody constant regions, proved to be efficient in both binding and ELISA-based ACE-2 blocking assays. We, therefore, further humanized the variable domains using the proprietary Qumanize algorithm (Icosagen Cell Factory OÜ, Estonia), which locates CDR and framework regions based on the WolfGuy antibody numbering scheme [33] and substitutes amino acids based on the closest germline and known human antibody sequences. As a result, we obtained the humanized antibody designated as ICO-hu104, with a humanization score > 85%, as evaluated using the IMGT/DomainGapAlign tool [34]. We determined the binding properties of the ICO-hu104 antibody by bio-layer interferometry using six different VoC S-protein trimers (S5A Fig). Humanized antibody ICO-hu104 bound to all tested S-protein trimers with high affinity, showing picomolar (10−10–10^−12^ M) Kd values (S5B Fig).

### Neutralizing potency of the humanized antibody ICO-hu104

**Fig 2:**
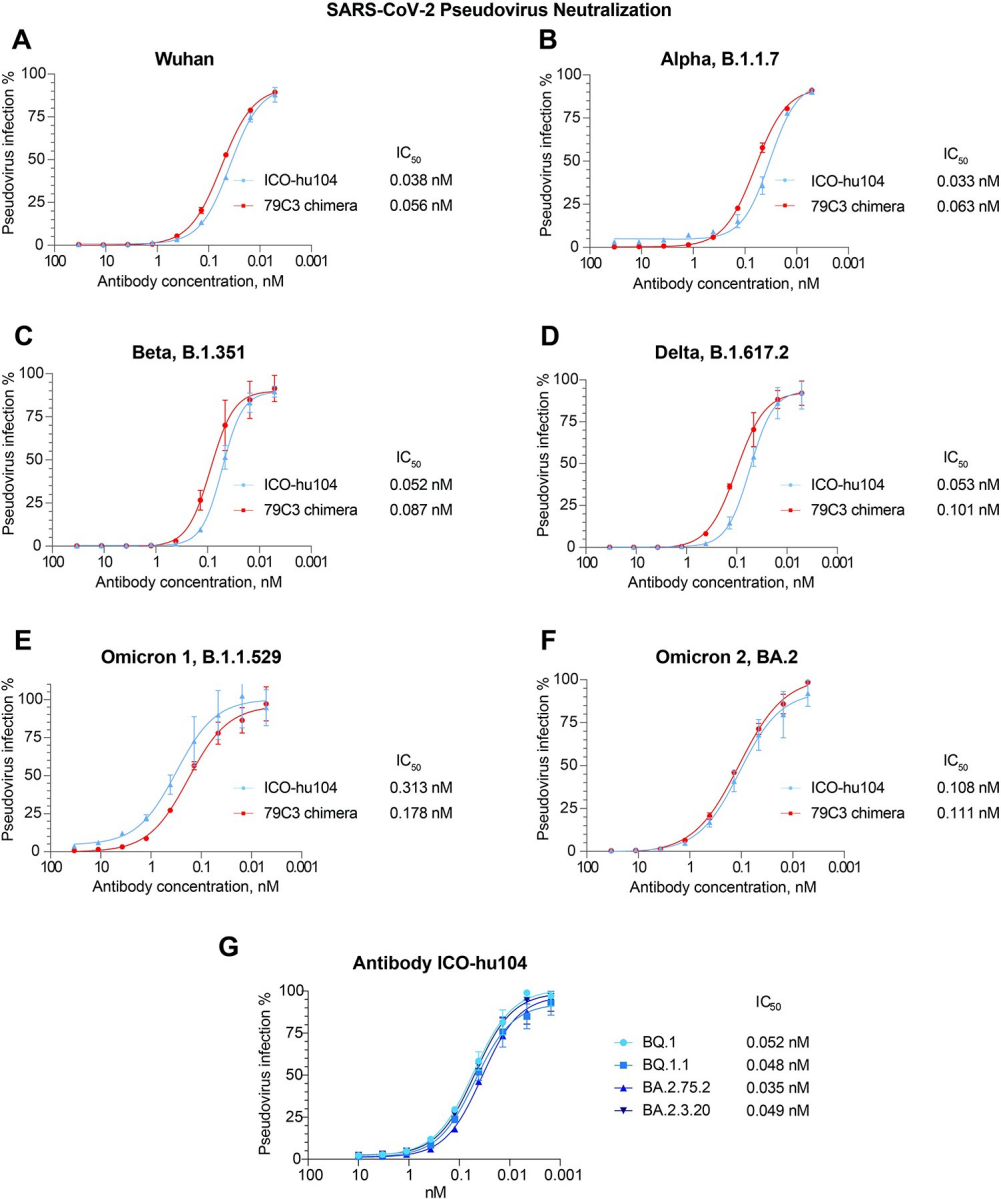
Chimeric and humanized variants of rabbit antibody 79C3 show wide neutralization activity against tested SARS-CoV-2 VoCs. **A-F**. Both the 79C3 chimeric antibody and ICO-hu104 humanized antibody show neutralizing activity against SARS-CoV-2 VoC Spike protein pseudoviruses in a pseudovirus assay. **G.** Antibody ICO-hu104 has maintained its pan-neutralizing activity for Omicron sub-variants as tested in a pseudovirus assay.

We assessed the pan-neutralizing activity of ICO-hu104 by performing a pseudovirus assay using a lentiviral pseudovirus system with S-protein proteins from the Wuhan, Alpha (B.1.1.7), Beta (B.1.351), Delta (B.1.617.2), Omicron (B.1.1.529), and Omicron 2 (BA.2) strains. We used the chimeric rabbit antibody as a reference to assess the direct effect of substitutions in the variable domains on neutralizing potency. The humanized antibody showed sub-nanomolar IC50 values for all six virus variants (Fig 2A–2F), with minimal differences from those of the chimeric antibody. The IC50 values for both the humanized and chimeric antibody for the assessed strains (excluding Omicron (B.1.1.529)), varied from 0.033 to 0.111 nM. Conversely, for Omicron (B.1.1.529), the chimeric antibody showed better neutralizing potency than the humanized antibody, with IC50 values of 0.178 nM and 0.313 nM, respectively. Compared to homologous (Wuhan) strain the IC50 value of ICO-hu104 was increased for all VoCs other than Alpha; the differences being minimal for Beta and Delta and maximal (approximately 8-fold) for Omicron. We later confirmed the neutralizing activity of antibody ICO-hu104 on Omicron subvariants spreading mostly later in 2022 (Fig 2G). Despite some reduction, the low IC50 values still reveal ICO-hu104 as pan-VoC neutralizing antibody.

Neutralizing activity of ICO-hu104 was also confirmed in a cytopathic effect assay with both Delta (B.1.617.2) and Omicron (B.1.1.529) strains. Respectively the IC100 values for Delta and Omicron strains varied respectively from 0.24 to 0.75 nM and from 0.7 to 1.12 nM.

### Nebulization of ICO-hu104 allows the delivery of functional antibody to the lower respiratory tract and provides antiviral protection

**Fig 3:**
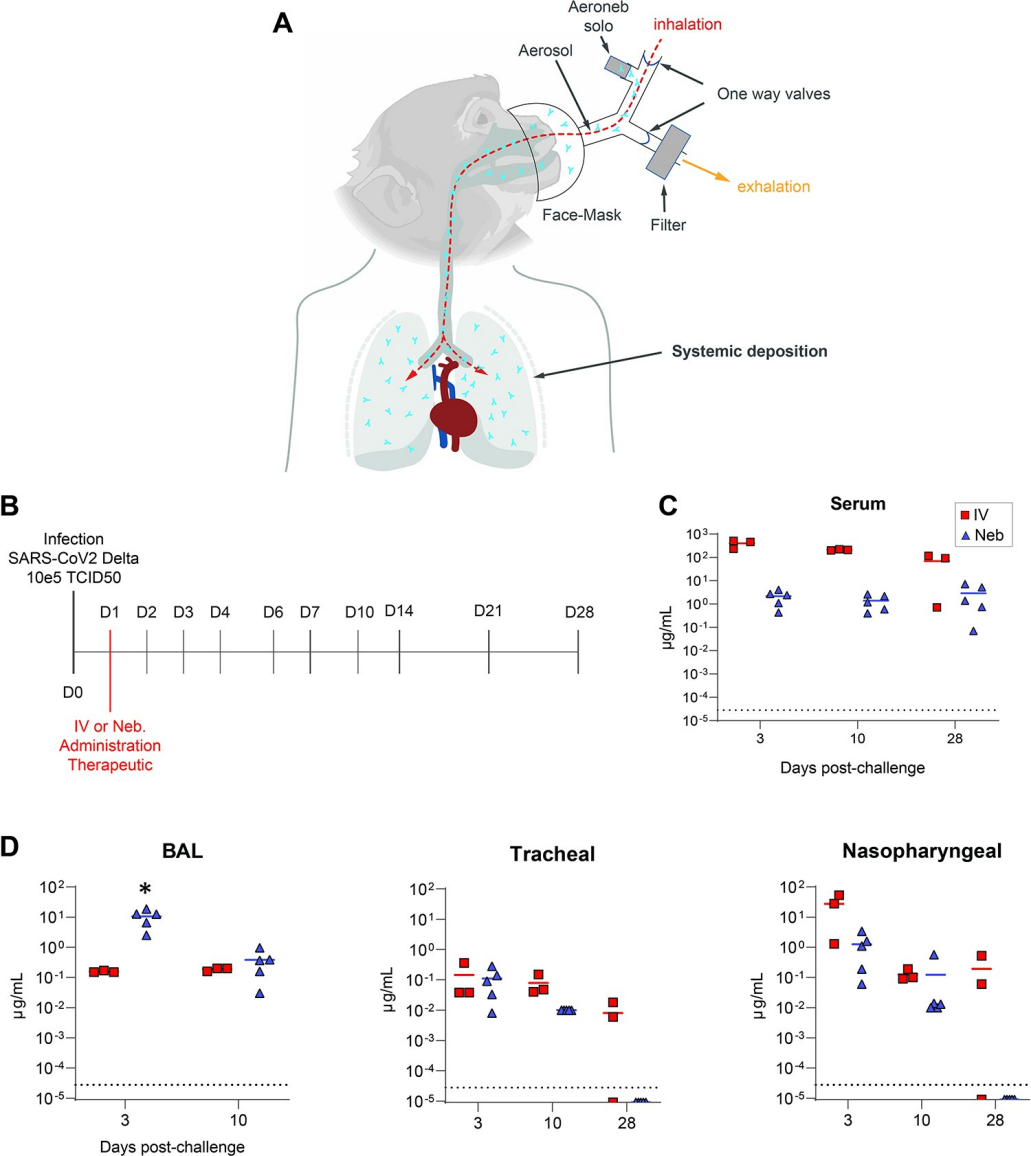
Antibody ICO-hu104 is detected in the serum and other locations after both intravenous administration and nebulization. **A.** Schematic representation of the antibody nebulization system adapted for cynomolgus macaques allowing the localized delivery of antibodies. Rhesus macaque head feature is an asset aquired from BioRender.com. **B.** Schematic representation of the pharmacology study protocol of non-human primates. Three groups (control [n = 2], intravenous administration [n = 3], and nebulized administration [n = 5]) were exposed to 10^5^ median tissue culture infectious dose (TCID50) of SARS-CoV-2 Delta strain (B.1.617.2) through the intranasal and intratracheal routes. Treated animals received 25 mg/kg of antibody ICO-hu104 one day after the challenge and were sampled over 28 days, as described. For the analysis, we added historical controls challenged under the same conditions (n = 10). **C.** Antibody ICO-hu104 serum concentrations (mean with SEM). Red represents intravenously administered antibody and blue nebulized antibody. The dotted line represents the LOQ. **D.** Antibody ICO-hu104 concentration (μg/mL) in heat-inactivated bronchoalveolar lavage (BAL) (left), tracheal fluids (middle), and nasopharyngeal fluids (right) (means ± SD). The dotted line represents the LOQ. **C. and D.** LOQ = 0,000028 μg/mL, LOD = 0,000009 μg/mL. Red squares represent intravenously administered antibody and blue triangles represent nebulized antibody. Mann-Whitney unpaired two-tailed t-test, *p < 0.05.

**Fig 4:**
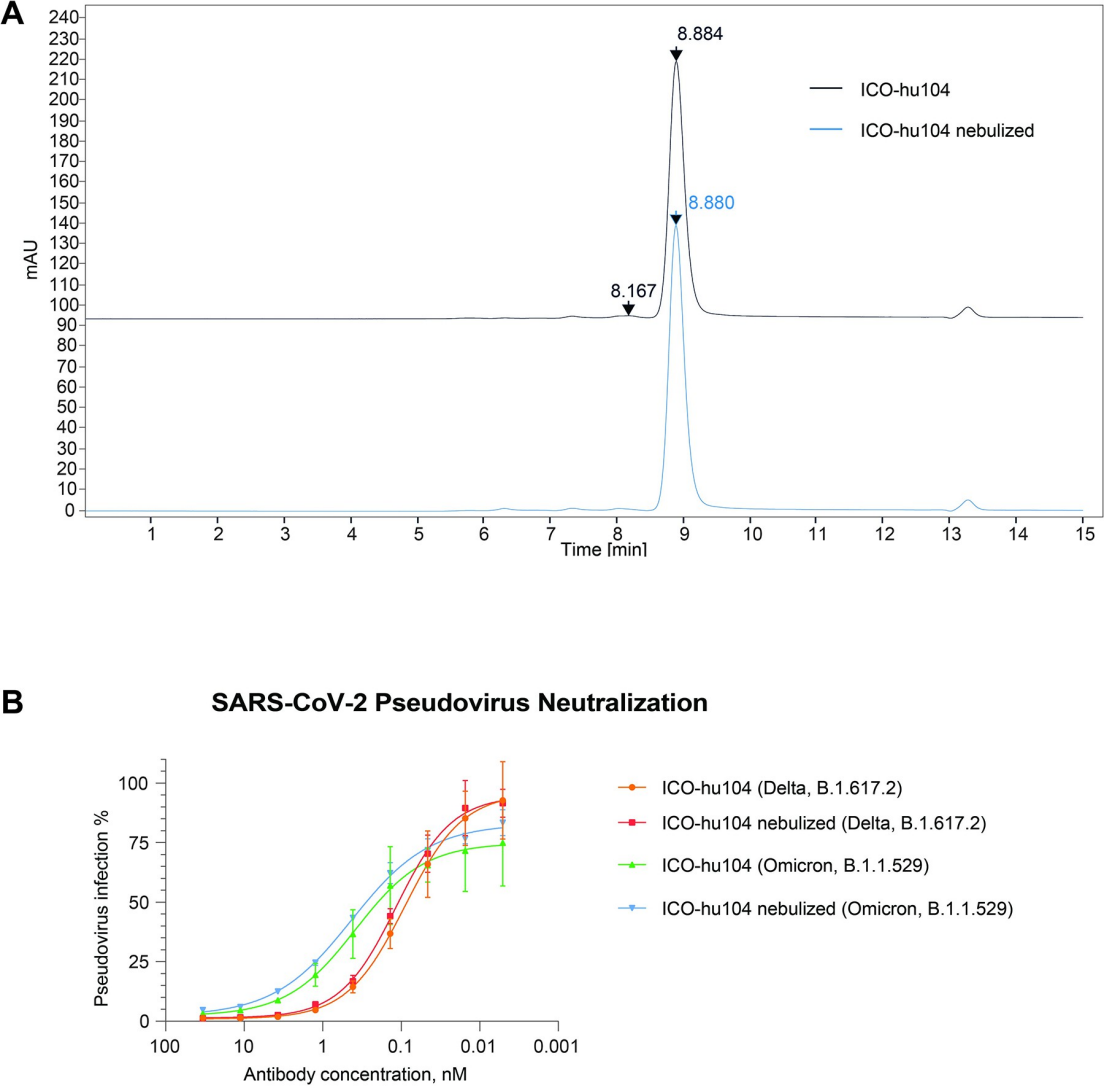
Antibody ICO-hu104 retains its integrity and function after nebulization. **A.** Analytical size-exclusion chromatography of the ICO-hu104 antibody before and after nebulization. **B.** Delta and Omicron Spike protein based pseudoviral neutralization assay of ICO-hu104 antibody pre- and post-nebulization.

Therapeutic antibodies against SARS-CoV-2 have proven to be effective in preventing the development of severe COVID-19 if administered at early stages of the infection. Furthermore, the rapid administration of neutralizing mAbs (NAbs) to case contacts who cannot benefit from vaccination can limit the spread of the virus. One limitation for the use of NAbs for prophylaxis or outpatient treatment lies in the cumbersome IV bolus procedures, which requires an extended period of time in a clinical setting. Rapid and easy delivery, such as that offered by Neb., can further broaden the impact of NAbs. We evaluated the efficacy of antibody Neb. by performing a viral challenge study and comparing the effect of nebulized and intravenously administered antibody on the viral load in a non-human primate model (NHP). Neb. of the humanized antibody by a vibrating mesh nebulizer (Aerogen Inc.) (Fig 3A) allowed the retention of antibody integrity and functionality, as demonstrated by analytical size exclusion chromatography (SEC) (Fig 4A) and pseudovirus neutralization assays (Fig 4B).

We tested the efficacy of ICO-hu104 delivered by Neb. using an Aeroneb Solo mesh Nebulizer with a Y-piece adapted to a breathing mask to enhance particle rebreathing (Fig 3A). Naïve animals (S6 Fig) were challenged on day 0 with 10^5^ median tissue culture infectious doses (TCID50) of the SARS-CoV-2 Delta strain (B.1.617.2) via combined intranasal and intratracheal routes using an experimental protocol we previously developed [35,36]. Then, the animals were treated intravenously (n = 3) or by the Neb. route (n = 5) 24 h after viral challenge with the nebulization of a single dose of 25 mg/kg of ICO-hu104 (Fig 3B). Twelve animals treated either with the Ab buffer (controls) or not receiving any treatment (historical controls) were used as controls.

Nuclear imaging is very frequently used in aerosol therapy to characterize aerosol distribution and calculate inhaled drug doses [37] and especially for monoclonal antibody nebulization [38]. [^18^F]-FDG nebulization prior to PET/CT imaging has been already implemented in macaques with generated aerosols of varying sizes [39]. A similar droplet size distribution of a PET/CT radiotracer ([^18^F]-FDG) versus the therapeutic monoclonal antibody (ICO-hu104) was validated with a cascade impactor (S7A Fig) allowing comparison of distribution. We used PET/CT to visualize and quantify deposited dose of [^18^F]-FDG and extrapolate to the ICO-hu104 deposited doses in different compartments of NHP respiratory tract (S7B Fig). The ICO-hu104 deposited dose was estimated in the whole respiratory tract, the upper and the lower respiratory tract using comparison with nebulized [^18^F]-FDG (S7C Fig). The median deposited dose was estimated at 7.0 mg/kg ±-SD (1.0) in the whole respiratory tract. The repartition was estimated at 4.7 mg/kg +±SD (1.1) in the upper respiratory tract and 2.4 mg/kg ±SD (0.6) in the lungs (lower respiratory tract).

The ICO-hu104 concentration was monitored during prolonged follow-up in blood and fluids and was detected in sera, as well as in upper and lower respiratory tract fluid samples, regardless of the administration route. IV route administration resulted in a 100-fold higher systemic concentration during the 28 days of follow-up, with a peak in the serum just after administration and high systemic concentrations still present 48 h later (430 μg/mL) (Fig 3C). In addition, 48 h after administration, the nasopharyngeal compartment of IV-treated animals showed higher concentrations of ICO-hu104, with a mean of 27.4 μg/mL ± SD (25.9) versus 1.3 μg/mL ± SD (1.3) for nebulized NHPs. The tracheal compartment also contained a significant amount of treatment Ab 48 h after administration. However, Neb. favored the biodistribution of ICO-hu104 to the lower respiratory tract, resulting, on average, in a concentration of 10.6 μg/mL ± 6.2 (SD) in BAL on day 3, whereas the i.v route resulted in only 0.16 μg/mL ± 0.01 of ICO-hu104 (Fig 3D).

**Fig 5:**
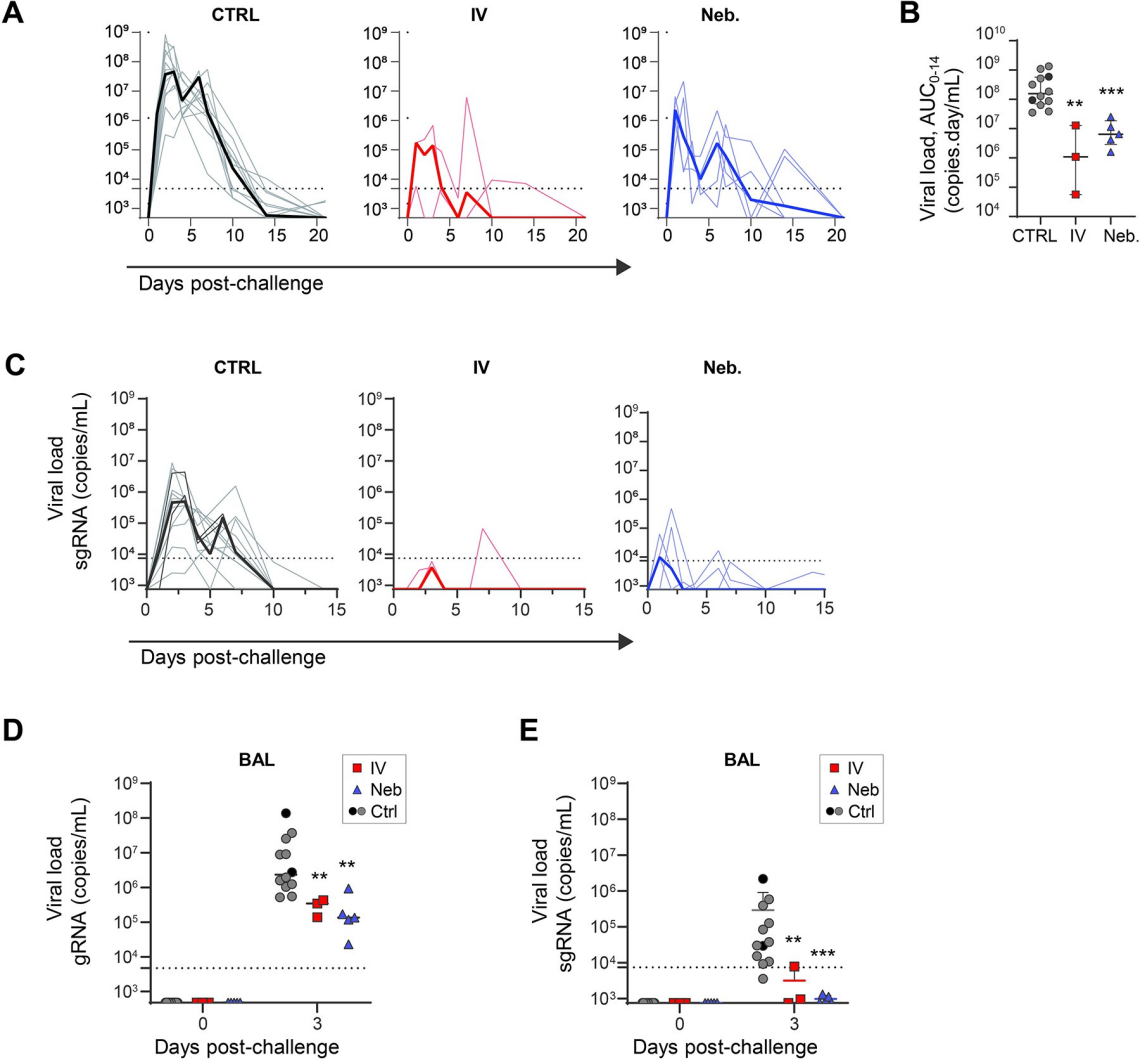
Antibody ICO-hu104 is capable of reducing the viral load in non-human primates through nebulization and intravenous administration. **A.** Genomic (g)RNA in nasopharyngeal fluids is expressed as viral copies per mL. Individual values are plotted by group. Median values are represented by the thick line. The dotted line represents the LOQ = 4760 copies/mL for gRNA (CTRL: control group; in black the extemporaneous controls, IV: intravenous treatment, Neb: Nebulization treatment, LOQ: limit of quantification). **B.** The area under the curve for data from days 0 to 14 (AUC_0-14_) of gRNA VL is shown for each individual (Kruskal-Wallis test p<0.0001 followed by Mann-Whitney unpaired two-tailed t-test: **p < 0.01, ***p < 0.001). Black circles represent the 2 extemporaneous control and the grey circles the historical controls, red the squares intravenous treatment group, and blue triangles the nebulization treatment group. **C.** Subgenomic (sg)RNA determined by RT-qPCR in nasopharyngeal fluids. Individual values are plotted by group. Median values are represented by the thick line. The dotted line represents the LOQ = 7490 copies/mL for sgRNA. Genomic RNA (**D**) and subgenomic RNA (**E**) viral loads determined by RT-qPCR of bronchoalveolar lavages (BAL). Individual values are plotted. The bars represent the median values for each group. Kruskal-Wallis test p<0.0001 followed by Mann-Whitney unpaired two-tailed t-test: p** < 0.01, p***< 0.001.

**Fig 6:**
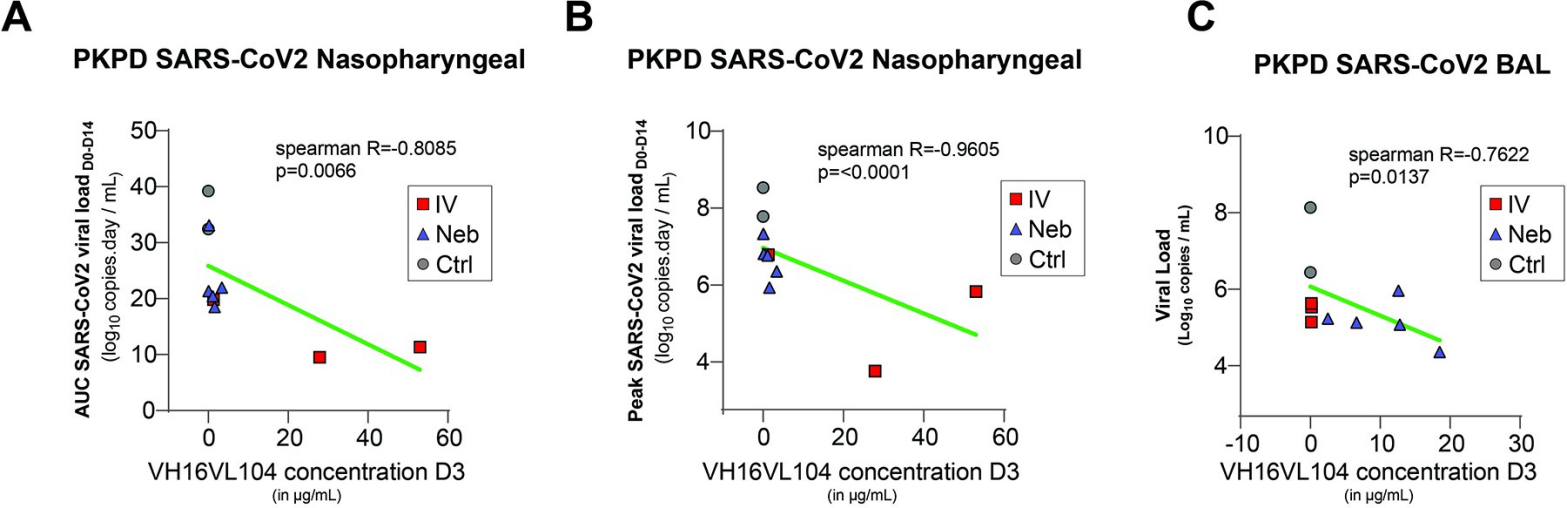
Antibody ICO-hu104 inhibits viral replication. Viral kinetic parameters (peak and AUC log10 viral load between 0 and 14 dpi) according to the concentration of ICO-hu104 IgG at day 3 in nasopharyngeal fluids with LOD of peak viral load at 2.68. Viral load at day 3 in BAL according to the concentration of ICO-hu104 IgG at day 3 in BAL. A Spearman correlation test was performed to assess the association between Ab concentration and viral kinetic parameters. Two-tailed p value is indicated. Grey: untreated; Red: IV.; Blue: Neb. NS: Nasopharyngeal.

We included 10 historical controls in addition to two simultaneous mock-nebulized controls, these historical controls were challenged with the same virus strain from the same virus stock at a similar dose. Following viral challenge with the SARS-CoV-2 Delta strain (B.1.617.2), the 12 control animals showed gRNA viral loads of 7.6x and 6.7x log_10_ copies per ml at 2 d.p.i. in nasopharyngeal and tracheal swabs, respectively (Figs 5A and S8A). We also assessed subgenomic (sg)RNA levels, with median values of 5.7x and 4.3x log_10_ copies per ml at 2 d.p.i. in nasopharyngeal and tracheal swabs, respectively (Figs 5C and S8C). At 3 d.p.i., viral loads were detected in the BAL, with a median value of 6.4x log_10_ copies per ml of gRNA and 4.5x log_10_ copies per ml of sgRNA. In comparison, at day 2 post-challenge (only day 1 post-treatment), IV- and Neb.-treated animals already showed a reduction of gRNA levels in nasopharyngeal swabs by 2.7x log_10_ (*p* < 0.01 compared to controls) and 2.1x log_10_ copies/mL (*p* < 0.05 compared to controls), respectively (Fig 5A). The reduction of nasopharyngeal viral load between control and treated animals was also highly significant at day 4 post-challenge (*p* < 0.01 for IV and *p* < 0.001 for nebulized). We observed the same trends in the tracheal compartment (S8A Fig). Comparison of the areas under the curve of the gRNA viral load confirmed that nasopharyngeal viral loads were lower in treated animals during the 14 days following exposure to the virus (Fig 5B) (*p* < 0.01 for IV and *p* < 0.001 for Neb). Although we observed a reduction in the area under the curve for tracheal fluids, we had too few animals to achieve statistical significance (S8B Fig). The sgRNA levels peaked between day 1 and day 3 post-challenge in the nasopharyngeal compartment for the various groups and showed a reduction in the viral load by 2.8x and 2.1x log_10_ for the IV and Neb. groups, respectively, relative to controls at day 2, corroborating our previous observations (Fig 5C). In the lower respiratory tract, the BAL viral load at 3 d.p.i. (Fig 5D and 5E) was significantly lower at day 3 post-challenge, as assessed by the level of gRNA (p < 0.01 for both treated groups) and sgRNA (p < 0.01 for the IV route and p < 0.001 for Neb.) (Fig 5E). We analysed the correlation between the Ab concentration at equilibrium and viral replication and showed that there is an effect of ICO-hu104 concentration at day 3 in nasopharyngeal fluids on nasopharyngeal AUC and peak viral load (p = 0.0066 and p<0.0001 respectively) (Fig 6A and 6B). The same results were observed in BAL fluid, with a negative correlation between ICO-hu104 concentration at day 3 and BAL viral loads at day 3 (p = 0.0137) (Fig 6C). Altogether, these data confirm that ICO-hu104 treatment inhibits SARS-CoV-2 replication in upper and lower respiratory tracts. In addition, we showed that the higher concentration of Ab in BAL after a Neb. treatment correlates with lower viral load in BAL. In parallel we performed a clinical follow-up of the challenged NHPs and measured some specific inflammatory cytokines and chemokine expression at the peak of virus replication (day 2 post-challenge) (S9 Fig). Antibody treatment tends to reduce these cytokine and chemokine expression with a significant reduction of IL15 and IL1RA in the IV. TT group. Biochemistry data collected during the acute phase of infection (S10 Fig) shows a transitory and moderate increase of ASAT and haptoglobin levels as reported previously in macaque model [40] and no negative impact of antibody treatment on this marker.

### Determination of the binding epitope of ICO-hu104

**Fig 7:**
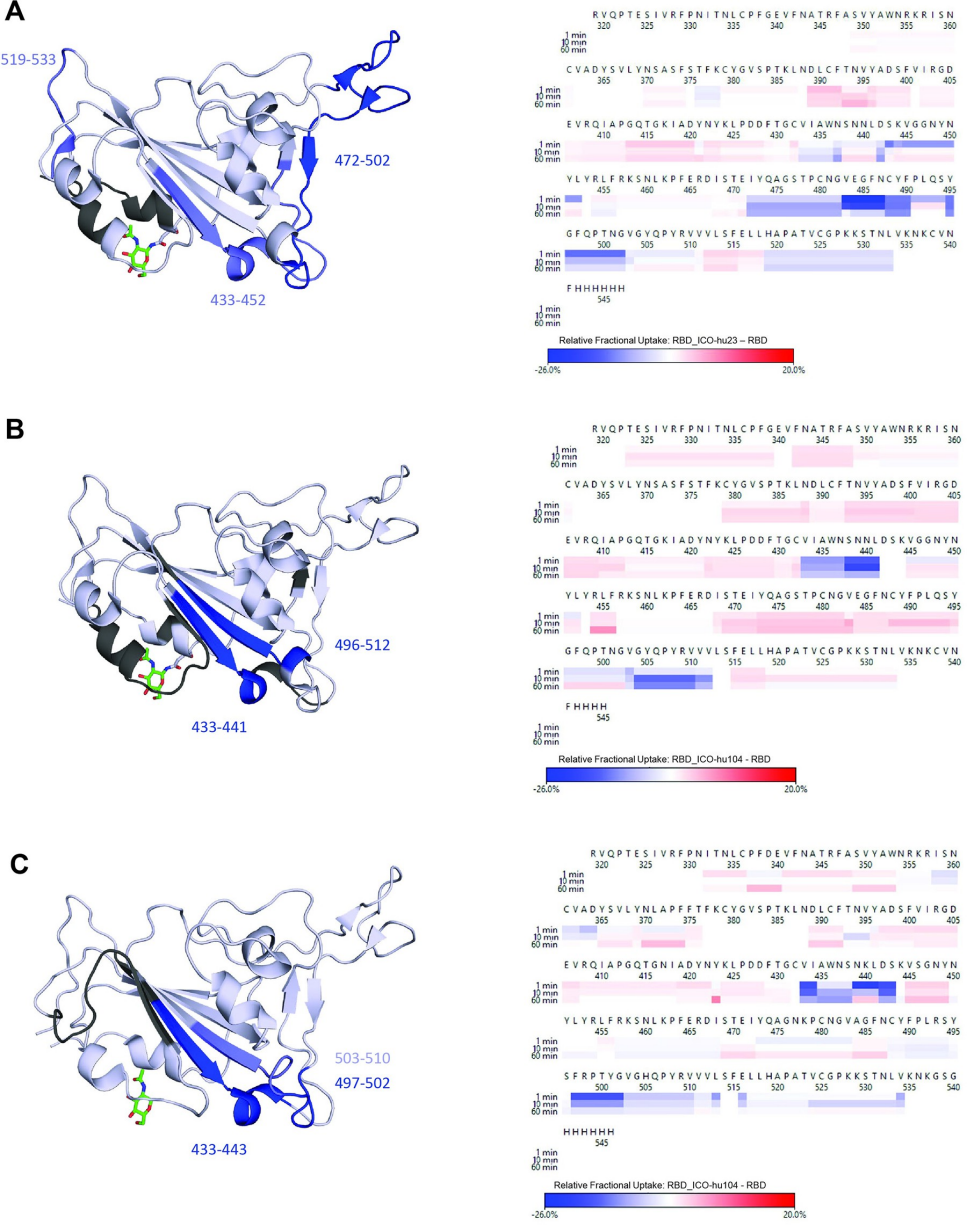
Differential HDX heatmap reveals epitope for ICO-hu23 and ICO-hu104. Heatmaps (right) show differential HDX uptake at 1, 10, and 60 min. The structures (left) illustrate the location of the epitopes in blue (summed relative fractional uptake difference > 20%) and weaker conformational changes in sky blue (5% <x < 20%). White areas indicate no relative fractional uptake and black areas indicate no sequence coverage. For the Wuhan RBD, PDB ID 6m0j was used and PDB ID 7wsk for the Omicron RBD. **A.** Wuhan RBD/ICO-hu23. **B.** Wuhan RBD/ ICO-hu104. **C.** Omicron RBD/ ICO-hu104.

We determined the binding epitope of the ICO-hu104 antibody by performing hydrogen-deuterium exchange mass-spectrometry analysis (Fig 7) using the Wuhan-selective antibody ICO-hu23 as a reference (Fig 7A). Differential HDX experiments were conducted at 1, 10, and 60 mins at room temperature on the RBD alone and a 1:1 RBD/antibody complex. For ICO-hu23, a sequence coverage of 77.5% and average redundancy of 4.59 was obtained for the Wuhan RBD, with no apparent binding to the Omicron RBD. For the Wuhan RBD, high magnitude decreases in deuterium uptake were observed within region 472–502, and moderate decreases in regions 433–452 and 519–533. This epitope is primarily situated within the disordered loops on the surface of the RBD that are known common ‘orthosteric’ sites for SARS-CoV-2 S-protein neutralizing antibody binding [41]. Notably, the 472–502 region includes six Omicron mutation sites (S477N, T478K, E484A, Q493R, G496S and N501Y), which is consistent with the lack of affinity of ICO-hu23 for the Omicron RBD.

For the pan-VOC neutralizing antibody ICO-hu104 (Fig 7B and 7C), a sequence coverage of 72.3% and 83.8% was obtained for the Wuhan RBD and Omicron RBDs, respectively, and a redundancy of 2.97 for both. For the Wuhan RBD, we observed large-magnitude HDX decreases for regions 433–441 and 505–512 (Fig 7B), and moderate decreases for region 496–504. The Omicron RBD exhibited strong differences in uptake within regions 433–443 and 497–502, with moderate responses for region 503–510 (Fig 7C). In both strains, this region corresponds to a unique epitope that lies within an exposed beta sheet that is further removed from the S-protein/ACE2 binding interface than the common ‘orthosteric’ sites. Consistent with the pan-binding/neutralizing properties of ICO-hu104, this region includes zero mutations to distinguish Wuhan from Omicron as antigens.

### Structural analysis of the Spike trimer-Fab ICO-hu23 interaction

To better understand the loss of function, we first used cryo-electron microscopy (cryo-EM) in combination with single particle analysis to study the interaction of the Spike trimer with Fab ICO-hu23. 3D classification of the particles revealed two different complexes with either two or three Fabs bound to each S-protein trimer. Refinement of the complex with three Fabs bound yielded a map at 3.3 Å resolution (S11 Fig, S1 Table). The resulting model showed that all Fabs were bound to the RBD in the down conformation. Most interactions were mediated by the ICO-hu23 heavy chains, with heavy-chain complementary-determining regions (CDRs) 1–3 recognizing the concave tip of the SARS-CoV-2 RBD. Compared to the core of the Spike trimer, the resolution around the ICO-hu23 variable domain region was considerably lower, indicating greater conformational dynamics in that region and the inability to accurately model the atomic details.

We then determined the crystal structure of the RBD in complex with Fab ICO-hu23 at 2.89 Å resolution to observe the interaction between the RBD and Fabs at atomic resolution (S12A Fig, S2 Table). The RBD was bound predominately to the Fab VH domain by an interface area of 655 Å^2^ and partially to the VL domain by an interface area of 128 Å^2^ (calculated using PISA [42]). The RBD formed several contacts with all three complementary determining regions (CDRs) and one additional loop of the VH, whereas it only formed a few contacts to aromatic residues of the VL (S12B and S12C Fig; for details see S3 Table).

The crystal structure showed several key residues of the SARS-CoV-2 RBD to be essential for the binding of Fab ICO-hu23. One central residue of the RBD is Glu-484, which formed polar contacts with the sidechains of three Fab VH residues, Tyr-33, Asn-52, and Ser-55 (S12B Fig and S3 Table). Several variants of SARS-CoV-2 have emerged over the last two years with mutations occurring in sites used by many identified SARS-CoV-2-neutralizing antibodies. When the negatively charged Glu-484 is replaced by a positively charged lysine in the Beta and Omicron VoCs, the ICO-hu23 antibody loses its neutralization capacity because the E484K variant disrupts the direct polar contacts, as well as the extended polar network (S12B and S12C Fig). Another key residue of the RBD is Phe-486, which was tucked into an aromatic pocket composed of two Fab VH residues, Trp-104 and His-100, and three Fab VL residues, Tyr-32, Tyr-34, and Tyr-93 (S12C Fig).

## Discussion

The SARS-CoV-2 pandemic is continuing with limited available therapeutic interventions. Monoclonal antibodies have proven to be an effective approach, providing a therapeutic impact by reducing the disease burden and reducing chances of hospitalization and death. However, the development of mAbs as therapeutics against viral infections such as SARS-CoV-2 is limited by cumbersome administration modes that limit the effective delivery of virus-neutralizing molecules to the site of infection. In addition, the dynamic nature of the SARS-CoV-2 S-protein has resulted in many clinical stage mAbs generated against viral variants of the early stage of the pandemic showing significantly decreased or no virus-neutralization capacity against the newly emerged Omicron and its subvariants [43,44]. Similarly, the first generation ICO-hu23 antibody, isolated here from a convalescent patient, demonstrated similar vulnerabilities with the emergence of different VoC’s, especially Beta and Omicron, which resulted in the loss of binding and virus-neutralizing capacity. To identify mAbs that could have highly potent pan-VoC-neutralizing capacity, we screened antibodies obtained from rabbits immunized with the original Wuhan variant S-protein against the Beta variant (B.1.351). The identified humanized ICO-hu104 antibody elicited potent binding to all tested VoC’s, with low picomolar binding affinities (< 1*10^−12^ M to 9.61 pM) towards the S-protein trimer. Furthermore, the ICO-hu104 antibody showed sub-nanomolar IC50 values in a pseudovirus neutralization assays against Wuhan, Alpha, Beta, Delta, Omicron and its multiple subvariants. In our efforts to identify the epitope of such a pan-VOC binding antibody, we performed HDX-MS analysis with the convalescent patient-derived ICO-hu23 and humanized ICO-hu104 antibodies. The epitope bound by ICO-hu23 determined by HDX-MS was in accordance with that identified in the obtained high resolution X-ray structure, showing binding to similar epitopes by many known class II antibodies [45]. HDX-MS analysis showed a high decrease in deuterium uptake in region 472–502, which is heavily mutated in Omicron VOCs, with six substitutions in this area, conferring the loss of binding. High resolution X-ray structural analysis identified the importance of S-protein residue Glu-484 for ICO-hu23 binding, which forms polar contacts with the sidechains of three VH residues of ICO-hu23. In comparison, the ICO-hu104 antibody showed high deuterium mass changes in region 433–443, which is distal from the common orthosteric binding site of ICO-hu23. This area includes an exposed beta sheet that has remained conserved throughout the original Wuhan virus and all tested variants. This indicates that the epitope bound by ICO-hu104 may be significantly more resistant to antigenic drift and shift and could allow antibodies directed against this epitope to retain their neutralizing efficacy longer, making such antibodies attractive lead candidates for further therapeutic development and characterization. Nevertheless, this does not indicate that mutations emerging in other regions of the Spike protein do not alter with the binding and neutralization efficacy of our antibody. Most of the clinically used antibodies have shown some or complete loss to the emerged SARS-CoV-2 variants [46,47], referring that the classical therapeutic monoclonal antibody efficacy against the SARS-CoV-2 virus is difficult to retain as the virus evolves while circulating in populations. Therefore, it seems inevitable that viral escape mutants could emerge for ICO-hu104 over time.

Therapeutic antibodies against multiple pathogens, including viruses, for the treatment and management of infections of the respiratory tract are becoming increasingly relevant [24]. Current mAb-delivery treatments have focused on systemic delivery, either via IV or subcutaneous delivery. Monoclonal antibodies have been shown to be transported from the blood to the lung in various animal models [25]. Although high systemic antibody levels can be obtained, the transport of the virus-neutralizing antibodies to the mucous membranes for protective and therapeutic purposes is dependent on FcRn receptors [48]. FcRn expression is restricted to the epithelial cells of the upper airways and alveolar macrophages in humans and macaques [26]. Although IgG can, thus, cross epithelial barriers by receptor-mediated transcytosis, mAb levels in serum are 500 to 10,000 times higher than in BAL after IV administration, even though the flow between compartments is bidirectional [27]. To reduce the overall dose, cost, and potential side effects associated with systemic delivery, aerosol therapy has been specifically developed for protein delivery to the lungs, including that of mAbs [24,28,49]. As SARS-CoV-2 virus enters through the upper respiratory tract and then diffuses into the airways [50], Neb. appears to be the optimal approach to maximize mAb concentrations in the upper respiratory tract and pulmonary lumen. Recent studies with inhalable remdesivir in NHP models and aerosol-delivered mAbs in hamster models have demonstrated more therapeutic potential than IV administration [51,52].

However, the setting up of this technique presents a number of challenges, such as an antibody-based formulation for aerosol therapy [53] and the choice of nebulizer to ensure the structural stability and neutralizing activity of the mAb. Among various nebulizers, vibrating mesh nebulizers are the most suitable devices for maintaining the physicochemical properties of Abs [28]. To test our compound in NHPs, we decided to adapt an Aeroneb solo nebulizer to a facemask equipped with unidirectional valves, allowing maximum aerosol deposition, as the macaques were anesthetized and breath spontaneously [54].

Antibody instability upon Neb. has been considered to be a potential limitation. However, such risks can be mitigated with formulation optimization [55]. Indeed, aggregation, measured by SEC analysis, and loss of virus-neutralization post-Neb was not detected for ICO-hu104. *In-vivo* delivery of inhaled ICO-hu104 antibody to NHPs via a clinically approved vibrating mesh nebulizer device resulted in broad distribution throughout both the upper and lower respiratory tracts, with significantly higher antibody levels measured in lung BAL. Importantly, inhalation-based delivery also resulted in systemic detection of the antibody in the bloodstream of the animals, indicating the possibility of the generation of systemic immunity via the inhalation-based delivery of antibodies. In the SARS-CoV-2 viral challenge, inhalation-based delivery resulted in a significant decrease in viral loads in the upper respiratory and lower respiratory tracts, corresponding to the lung compartment.

Several mAbs have been clinically evaluated as inhalation-delivered aerosols, with good preliminary safety and tolerability profiles. Increasing the accessibility to such antibody-based drugs through rapid and non-invasive inhalation approaches could have a significant impact in reducing the transmission, morbidity, and mortality of SARS-CoV-2.

## Materials and methods

### Ethics statement

Cynomolgus macaques (*Macaca fascicularis*), aged 2.5 to 4 years, body weight 2.9 to 4.4 kg (6 females, 4 males), originating from Mauritian AAALAC certified breeding centers were used in this study. All animals were housed in the IDMIT infrastructure facilities (CEA, Fontenay-aux-Roses) under BSL-3 containment (animal facility authorization D92-032-02, Prefecture des Hauts de Seine, France) and in compliance with European Directive 2010/63/EU, French regulations, and the Standards for Human Care and Use of Laboratory Animals of the Office for Laboratory Animal Welfare (OLAW, assurance number A5826-01, United States). The protocols were approved by the institutional ethics committee “Comité d’Ethique en Expérimentation Animale du Commissariat à l’Energie Atomique et aux Energies Alternatives” (CEtEA n° 44) under statement number A20_066. The study was authorized by the ‘Research, Innovation and Education Ministry’ under registration number (APAFIS#29191–2021011811505374 v1). All information on the ethics committee is available at https://cache.media.enseignementsup-recherche.gouv.fr/file/utilisation_des_animaux_fins_scientifiques/22/1/comiteethiqueea17_juin2013_257221.pdf.

### Protein preparation

RBD (Wuhan, S-protein amino acids 319–541; Icosagen (www.icosagen.com) catalog number P-307-100, name SARS-CoV-2 S1 RBD2), Omicron RBD (Omicron (B.1.1.529), S-protein amino acids 319–537; Icosagen catalog number P-367-100, name SARS-CoV-2 Spike RBD3 Omicron (B.1.1.529)), Spike S1 (S-protein amino acids 14–681; Icosagen catalog number P-305-100) and ACE2 (ACE2 amino acids 20–742; Icosagen catalog number P-302-100) were produced in CHO cells as 6xHis-tagged proteins and purified by Ni-NTA affinity chromatography.

Spike trimers (amino acids 14–1211; Icosagen catalog number provided for each mutant) used in the ELISA-based ACE2 blocking assays, binding assays, the bio-layer interferometry assay, and cryo-EM were designed as described in Wrapp et al. 2020, with a silenced furin cleavage site and a T4 fibritin trimerization motif in the C-terminus [56,57]. Instead of the HRVC3 protease cleavage site, a TwinStrepTag and 8xHisTag, GSG-linker, and 6xHisTag were used. Spike trimers were produced in modified CHO cell lines and purified by Ni-NTA affinity purification. The catalog numbers are as follows: Trimeric Spike Omicron 2 (BA.2): Icosagen catalog number P-376-100, Trimeric Spike Omicron (B.1.1.529): Icosagen catalog number P-369-100, Trimeric Spike Delta (B.1.617.2): Icosagen catalog number P-353-100, Trimeric Spike Beta (B.1.315) (South Africa VOC 501.V2: Icosagen catalog number P-316-100, Trimeric Spike Alpha (UK VOC 202012/01): Icosagen catalog number P-317-100), Trimeric Spike Wuhan: Icosagen catalog number P-309-100.

### Isolation of S1/RBD-binding human monoclonal antibodies using HybriFree technology

Monoclonal antibodies recognizing SARS-Cov-2 S1 protein were isolated using HybriFree technology [30]. Briefly, frozen PBMCs were thawed, washed, and collected in 10 ml RPMI1640 supplemented with penicillin/streptomycin (Pen-Strep) and 10% heat-inactivated fetal bovine serum (FBS). Then, the cells were seeded into a 100 mm cell-culture dish and incubated ~1 h at 37°C in an 8% CO_2_ atmosphere. Free-floating cells (the fraction enriched for B-cells and separated from plastic-adherent cells, e.g., macrophages) were collected, the viable cell count determined, and the cells transferred into the capture medium (RPMI1640 supplemented with 0.5% bovine serum albumin (BSA) and 0.1% NaN_3_).

For the capture or panning of B-cells that expressed SARS-CoV-2 S1 protein specific antibodies on their surface, MaxiSorp surface 96-well plates (Thermo Fisher Scientific, catalog number: 44-2404-2) were coated with the recombinant S1 subunit of the S-protein or RBD domain (amino acids 319–541) of the S1 subunit and blocked for 1 h with 2% BSA in phosphate-buffered saline (PBS). One hundred microliters of cell suspension containing 1 x 10^4^ live cells in capture medium was loaded into a single well. The plate was centrifuged (200 × g, 5 min) and incubated for 45 to 60 min. The medium was discarded and loosely attached cells removed by washing 4 to 5 times with PBS. Finally, the plastic-bound cells were lysed and subjected to total RNA isolation and cDNA synthesis using oligo-T primers. cDNA synthesis was performed using the SuperScript IV First-Strand Synthesis System (Thermo Fischer Scientific, catalog number: 18091050) and oligo d(T)_20_ primer supplied with the kit.

The cDNAs of the antibody VH and VL regions were amplified using cocktails of forward primers that bind to FR1 regions of VH or VL_kappa and VL_lambda light-chain coding sequences and were designed to maximally cover the variability of the human VH and VL sequences. In each reaction, a single reverse primer was used that binds to the beginning of the constant region-encoding sequence of the human IgG heavy chains, kappa light chain, or lambda light chain, respectively. The sequences of the primers used are as in Kivi et al. [30].

VH and VL PCR products retrieved from the same capture reaction were cloned into an expression vector using the ligase-independent cloning strategy as described in Kivi et al. [30].

The efficiency of the antigen-specific IgG reconstruction from the VH and VL combinations was initially analyzed via the transfection of library pools. The DNA was transfected into the Chinese hamster ovary cell line CHOEBNALT85 (Icosagen Cell Factory, Estonia) and 48 to 72 h later the culture supernatants were assessed by ELISA for the secretion of IgG molecules that specifically recognize the S1 protein of SARS-CoV-2, thus indicating the presence of the desired VH/VL combinations in the library.

Next, the library pools that showed clearly positive signals were split into individual clones by back-transformation into competent *E*. *coli* cells and picking individual bacterial colonies, each containing a single type of plasmid with a unique VH and VL combination. Then, the determination of specific S1-binding by ELISA was repeated using the supernatants of CHO cells transfected with plasmid DNA preparations derived from single clones instead of library pools. Finally, the VH and VL sequences were identified by sequencing the VH and VL insertions of the positive plasmid clones.

### Isolation of S1/RBD-binding rabbit monoclonal antibodies using HybriFree technology

Monoclonal antibodies recognizing SARS-Cov-2 S1 protein were isolated using HybriFree technology [30]. Briefly, approximately five-month-old New Zealand rabbits were initially immunized by two subscapular injections with the RBD of the S1 protein of the SARS-CoV-2 virus Wuhan isolate. The response was boosted by an IV injection of 0.1 mg of the same protein. After confirmation of an antigen-specific antibody response in the serum, spleens were collected 2 to 4 days after the final immunization. The animals were anesthetized and the spleens removed and stored on ice until treatment (within one hour). For the preparation of a cell homogenate, the spleen was homogenized in ice-cold PBS using a 40-μm cell dissociation sieve. Cells were precipitated and frozen in 1 ml cryovials using ice-cold freezing medium with 10% DMSO (Sigma Aldrich, catalog number: D8418) in heat inactivated FBS (Gibco, catalog number: A3160802). For antibody isolation, the frozen spleen-cell suspension was thawed, washed, and collected in 10 ml RPMI1640 supplemented with Pen-Strep and 10% heat-inactivated FBS. Then, the cells were seeded into a 100-mm cell culture dish and incubated ~1 h at 37°C in an 8% CO_2_ atmosphere. Free-floating cells (the fraction enriched for B-cells and separated from plastic-adherent cells, e.g., macrophages) were collected, the viable cell count determined, and the cells transferred into the capture medium (RPMI1640 supplemented with 0.5% BSA (Pan Biotech, catalog number: P06-1391500) and 0.1% NaN_3_).

For the capture or panning of the B cells that express SARS-CoV-2 S1 protein-specific antibodies on their surface, MaxiSorp surface 96-well plates were coated with the recombinant S1 subunit of the S-protein or RBD domain (amino acids 319–541) of S1 and blocked for 1 h with 2% BSA in PBS. One hundred microliters of cell suspension containing 1 x 10^4^ live cells in capture medium was transferred into a single well. The plate was centrifuged (200 × g, 5 min) and incubated for 45 to 60 min. The medium was discarded, and loosely attached cells removed by washing 4 to 5 times with PBS. Finally, the plastic-bound cells were lysed and subjected to total RNA isolation and cDNA synthesis using oligo-T primer. The cDNA synthesis was performed using the SuperScript IV First-Strand Synthesis System and oligo d(T)20 primer supplied with the kit. The proprietary primers used for the cDNA amplification of the antibody VH and VL regions and construction of the libraries expressing intact rabbit IgG molecules were designed using rabbit sequences stored at IMGT [58] and to cover the variability of the VH and VL sequences.

VH and VL PCR products retrieved from the same capture reaction were cloned into an expression vector using the ligase-independent cloning strategy as described in Kivi et al. [30].

The clones were analyzed as described for the isolation of human mAbs.

### SARS-CoV-2-induced cytopathic effect neutralization assay

Three-fold serial dilutions of purified antibodies were made using virus-growth medium. Medium without the preparation was used as a positive control for infection. The dilutions were performed in a 96-well plate in duplicate. Then, 100 plaque-forming units (pfu) of the Estonian isolate 3542 (S-protein sequence identical to the reference genome in Wu et al. 2020), Delta (B.1.617.2) and Omicron (B.1.1.529) SARS-CoV-2 strain were added to each well and the sample incubated for 1 h at 37°C. Next, 4 x 10^4^ Vero E6 cells were added to each well. The calculated multiplicity of infection was 0.0025 pfu/cell. The plates were incubated for four days at 37°C in a humid environment before evaluation of the cytopathic effect caused by viral infection. The same number of cells was grown without the virus as a negative control. After incubation, the cytopathic effect was evaluated microscopically. The experiment was performed in triplicate.

### SARS-CoV-2 Virus neutralization

Approximately 4 × 10^4^ Vero-E6 cells were seeded per well in 96-well plates. The cells were grown for 24 h in DMEM supplemented with 10% FBS and Pen-Strep. Antibody samples were prepared in three-fold dilutions at seven different concentrations, starting from 2 μg/mL, in VGM containing 0.2% BSA and Pen–Strep in DMEM. Virus strain hCoV-19/Norway/Trondheim-S15/2020 was added to achieve a multiplicity of infection of 0.1 and the cultures incubated for 1 h at 37°C. DMSO (0.1%) was added to the control wells. The Vero-E6 cells were overlaid with a VGM-containing mixture of the virus and convalescent serum. After 72 h, the medium was removed and a CellTiter-Glo assay (Promega, Oslo, Norway) was performed to measure cell viability.

### Syrian golden hamster viral challenge

Animal housing and experimental procedures were conducted according to the French and European

Regulations and the National Research Council Guide for the Care and Use of Laboratory Animals.

The animal BSL3 facility is authorized by the French authorities (Agreement N° D92-032-02). All animal procedures used in the current study were submitted to the Institutional Animal Care and Use Committee of the French Alternative Energies and Atomic Energy Commission (CEA) approved by the French authorities (CETEA DSV–n° 44).

SARS-CoV-2 strain “Slovakia/SK-BMC5/2020”, originally provided by the European Virus Archive global (EVAg), produced and titered by Oncodesign on VeroE6/TMPRSS2 cells, was used to infect the hamsters. Eight female Syrian golden hamsters per study group were used.

The viral load in each lung was quantified by RT-qPCR in duplicate using the IP4 gene (based on the protocol published by the French Pasteur Institute, listed by the WHO: https://www.who.int/docs/default-source/coronaviruse/real-time-rt-pcr-assays-for-the-detection-of-sars-cov-2-institut-pasteur-paris.pdf).

The extraction of viral RNA was performed using the QIAamp Viral RNA Mini Kit (Qiagen). Complete RT-PCR was run using the SuperScript III One-Step qRT-PCR System kit (cat. 1732–020, Life Technologies) with primers and RT-PCR conditions targeting the IP4 gene.

### Cell lines

For the development of ACE2-overexpressing HEK293 cell lines, human ACE2 receptor cDNA was excised from plasmid pLV-ACE2 using PsiI/AleI enzymes and the 7,405 bp fragment purified by agarose gel electrophoresis. HEK293 cells were transfected with 2 μg of the purified ACE2 DNA fragment by electroporation and 48 h later, hygromycin was added to the media at a final concentration of 200 μg/ml. Cells were grown under hygromycin selection for approximately two weeks.

### Pseudovirus assay

This pseudovirus assay is based on a lentivirus-pseudoviral system, where the infection of cells and integration of proviral DNA is dependent on Spike pseudotyping of pseudovirus. For the production of pseudovirus, 5 × 10^5^ HEK-293 cells were seeded per well in six-well plates. The cells were grown for 24 h in DMEM supplemented with 10% FBS and Pen-Strep. The next day, cells were transfected with 1 μg plasmid pNL4-3 [59] and 60 ng of plasmid encoding SARS-CoV-2 wildtype Wuhan Spike or a Spike variant. Transfection was carried out using Lipofectamine 2000 (Thermo Fischer Scientific, catalog number: 11668019) or Lipofectamine 3000 (Thermo Fischer Scientific, catalog number: L3000015) according to the manufacturer’s protocol (Thermo Fisher Scientific, US). Plasmids encoding the S-protein had a FLAG-tag at the N-terminus and a 19 amino-acid deletion at the C-terminus. Transfected cells were incubated for 72 h at 37°C in 5% CO_2_. HEK-293 cells overexpressing the human ACE2-receptor were resuspended in DMEM supplemented with 10% FBS and Pen-Strep and seeded at 1 × 10^4^ cells per well into white clear-bottom 96-well plates. After 72 h, supernatants were collected and filtered through 0.45 μm filters and used immediately or frozen at -80°C. Antibodies were prepared in the virus supernatant in three-fold serial dilutions at nine different concentrations, with a final column containing no antibody used as a control (enabling 100% infection). HEK-293 cells overexpressing hACE2 were infected overnight with virus supernatant containing the antibody dilutions and the medium was changed the next day. Steady-Glo Luciferase (Promega) activity was measured 72 h later using a GloMax reader. All luciferase activity was analyzed in relation with the positive control (e.g. no antibody added, considered as 100% of infection).

### ELISA assays

#### SARS-CoV-2 neutralizing antibody ELISA

The capacity of the antibodies to inhibit the binding of ACE2 (angiotensin-converting enzyme) to the coated SARS-CoV-2 Spike trimer was determined using a competition ELISA (enzyme-linked immunosorbent assay). The wells of Nunc MaxiSorp flat-bottom 96-well plates (Thermo Fischer Scientific, catalog number: 468667) were coated with purified SARS-CoV-2 Spike trimer (Icosagen Cell Factory, catalog number: P-309-100) at 2.5 μg/mL in PBS overnight at 4°C and blocked with a PBS solution containing 1% bovine plasma albumin (BPLA; Roche, catalog number: 11726536103), 0.1% ProClin 300 (Sigma-Aldrich, catalog number: 48914-U), and 2% sucrose (Serva, catalog number: 35580.02) for 1 h at room temperature (RT). The samples (purified mAbs) were diluted in assay buffer (0.5% BPLA, 0.1% ProClin 300, 2% sucrose in PBS) and added at a 1:2 dilution to the blocked plate, with a starting concentration of 16 μg/mL in the final volume of 50 μL, and pre-incubated for 20 min at RT at 450 rpm. Three wells on each 96-well plate contained assay buffer containing no antibodies as a negative control (NC). The enzyme complex (50 μL), consisting of biotinylated (EZ-Link NHS-PEG4-Biotin; Thermo Scientific; catalog number: 21955) ACE2-hFc (Icosagen Cell Factory, catalog number: P-308-100; 0.5 μg/mL) and Pierce High Sensitivity Streptavidin HRP (horse radish peroxidase) (Thermo Fischer Scientific, catalog number: 21132; 0.02 μg/mL), was added to the pre-incubated plates and the plates incubated for 30 min at RT at 450 rpm. The ACE2 in this enzyme complex competes with the antibodies to the specific receptor binding site (RBD) on the coated S-protein. Colorimetric development was performed using 3,3’,5,5’-tetramethylbenzidine VII substrate (Biopanda Diagnostics, catalog number: TMB-S-004). The reaction was stopped using 0.5 mol/L H_2_SO_4_ and the absorbance was measured at 450 nm. The optical density (OD) values of the measured samples were divided by the mean value of the three repeats of the NC to obtain the relative OD values. Relative OD values ≥ 0.75 were considered to show no neutralization, as the conjugated ACE2 could still bind to the RBD site. Relative OD values < 0.75 were considered to indicate neutralization activity of the analyzed antibody, as the antibodies were able to bind to the coated S-protein and thus hinder the binding of ACE2 to the RBD.

#### Spike trimer-binding ELISA

The capacity of the antibodies to bind Spike trimer protein of SARS-CoV-2 was determined by ELISA. The wells of Nunc MaxiSorp flat-bottom 96-well plates were coated with purified SARS-CoV-2 Spike trimer protein (Icosagen Cell Factory, catalog number: P-309-100) at 1 μg/mL in PBS overnight at 4°C and blocked with a PBS solution containing 2% BSA, 0.05% Tween 20 (Carl Roth, catalog number: 9127.1) for 1 h at RT. The samples (purified mAbs) were diluted in assay buffer (1% BSA, 0.05% Tween 20 in PBS) and added to the blocked plate in a final volume of 100 μL and incubated for 1 h at RT at 450 rpm. Secondary HRP-conjugated goat anti-human IgG antibody (Invitrogen, catalog number: A18823) was diluted in assay buffer at 1:10 000, added to the washed plate, and the plate incubated for 30 min at RT at 450 rpm. Colorimetric development was performed using 3,3’,5,5’-tetramethylbenzidine VII substrate. The reaction was stopped using 0.5 mol/L H_2_SO_4_ and absorbance was measured at 450 nm. Half-maximal (EC50) values were calculated using the Fourth Party Logistic Model (4PL).

### Bio-layer interferometry

Antibody binding kinetics were measured by bio-layer interferometry. All antibody and antigen dilutions for the assay were prepared in a kinetics buffer containing 0.1% BSA, 0.02% Tween 20, and 0.01% Proclin 300 in PBS. Biosensors were regenerated using 0.1M glycine buffer (pH = 1.7) and antibody and antigen dilutions were prepared in black flat-bottom 96-well plates (Greiner, catalog number: 655209). Purified wildtype and mutant Spike trimers were used as antigens at five different concentrations– 100 nM, 50 nM, 25 nM, 12.5 nM, and 6.75 nM. Octet Protein A (ProA) Biosensors (Sartorius, catalog number: 18–5010) were incubated for at least 10 min in 200 μl kinetics buffer before the assay. Biosensors were regenerated by three washes of 5 s with glycine buffer, followed by 5 s of neutralization in kinetics buffer. Sensors were then incubated in kinetics buffer for 1 min to establish a baseline, the antibody was added, and the plate incubated for 1 min, and then incubated again in the kinetics buffer to wash off the unbound antibody. Sensors were incubated for 5 min in the antigen solution to measure the association (k_ON_) and then for 10 min in kinetics buffer to measure the dissociation (k_OFF_). These steps were repeated for all antigens and the sensors were regenerated after each round.

### Analytical size-exclusion chromatography

Analytical size-exclusion chromatography (aSEC) was carried out to determine the monomeric content of nebulized and non-nebulized mAb samples. A 1-μg sample was loaded onto a Waters Biosuite High Resolution SEC Column (250 Å, 4 μm, 4.6 mm x 300 mm; Waters, catalog number: 186002162), with a BioSuite SEC Guard Column (5 μm, 6 mm x 40 mm; Waters, catalog number: 186002167). The analysis buffer consisted of 0.2 M potassium phosphate and 0.25 M KCl (pH = 6.2) in PBS. Absorbance of the flow was monitored using a UV-detector at 280 nm.

### Hydrogen deuterium exchange mass spectrometry epitope mapping

Hydrogen-deuterium exchange was carried out using the commercial Waters HDX system. Briefly: samples were mixed and injected in a labeling time-randomized order by a PAL3 Autosampler followed by UPLC separation and mass spectrometry analysis using a Waters M-Class ACQUITY UPLC with peptides detected on a Waters Select Series Cyclic IMS Mass Spectrometer. Labeling times of 1, 10, and 60 min at RT were used, followed by quenching (7.5 M guanidine hydrochloride and 0.5M TCEP) at 0°C and digestion using an Enzymate BEH Pepsin column. The peptides were reverse-phase separated using an ACQUITY UPLC BEH C18 column. Peptide identification was carried out using HDMSe with CID fragmentation in the transfer cell and ProteinLynx Global Server (PLGS), followed by HDX analysis using DynamX. Structures were visualized using PyMOL 2.5.

### Cryo-EM sample preparation, data collection, and data processing

The purified Spike trimer (Wuhan) was aliquoted, flash frozen, and stored at -80°C. Each aliquot was thawed and incubated at 37°C for five days before use to ensure correct folding of the cold-sensitive protein [60]. Then, the Spike trimer was mixed with a six-fold molar excess of Fab ICO-hu23 and incubated for 30 min at RT. The Spike trimer–Fab ICO-hu23 complex was purified by size-exclusion chromatography on a Superdex 200 increase 5/150 GL column in 20 mM Tris pH 8.0 and 200 mM NaCl. Peak fractions were collected for electron microscopy studies.

The Spike trimer–Fab ICO-hu23 complex (3.5 μL at 0.38 mg/ml) was loaded onto a freshly glow-discharged C-flat 2/1 holey carbon grid (300 mesh, Protochips) and incubated for 30 s. Excess protein was blotted away for 6 s with blot force 1 prior to plunge-freezing into liquid ethane using a Vitrobot Mark IV (ThermoFisher Scientific) at 100% humidity and 25°C.

Data were acquired at the Facility for Electron Microscopy Research of McGill University Montreal on a FEI Titan Krios transmission electron microscope operated at 300 kV and equipped with a Gatan K3 direct electron detector and a BioQuantum imaging filter. In total, 10,304 movies were collected at a nominal magnification of 105,000x with a pixel size of 0.855 Å. A total dose of 80 e^-^/Å^2^ was fractionated over 40 frames for each movie and a defocus range of -1.25 to -2.50 μm was used.

Data processing was first carried out using CryoSPARC [61]. The movies were aligned, motion corrected, and the CTF parameters estimated. Particles were picked from 200 micrographs to create a template for particle picking for the rest of the dataset. A box size of 540 pixels was used for particle extraction and all particles were subsequently subjected to several rounds of 2D classification. In total, 120,000 particles were selected and used for ab-initio reconstruction. The same set of particles was exported to Relion [62] and subjected to 3D classification. The best class with three Fabs bound (~36,000 particles) was subjected to CTF and 3D refinement (with symmetry C3 applied) in Relion and then exported back to CryoSPARC for a round of non-uniform refinement in symmetry C3.

### Cryo-EM model building and analysis

UCSF Chimera [63] and Coot [64] were used to fit atomic models into the cryoEM maps. PDB ID 6vxx was used as a starting model for the Spike trimer, PDB ID 6m0j was used for the RBD, and Fab variable domains were generated using the SWISS-MODEL protein structure homology-modelling server [65]. The models were then refined using Refmac5 [66] and validated using Molprobity [67].

### RBD–Fab ICO-hu23 complex crystallization and crystal harvesting

The complex was formed by mixing SARS-CoV-2 RBD and Fab ICO-hu23 in a 1:2 molar ratio at RT overnight. The protein complex was purified using a Superdex 200 10/300 GL column (GE Healthcare) in buffer containing 150 mM NaCl and 20 mM Tris at pH 7.5. The complex was concentrated to 10 mg/mL using a 10 kDa concentrator (Amicon) and screened using Nextal JCSG Core I-IV Suites crystallization kits in standard 96-well sitting-drop plates (Hampton Research) using a 1:1 ratio of protein to precipitant (1.4 μL drop total) with 100 μL of precipitant in the well. Positive hits were then optimized using standard 24-well sitting-drop crystallization trays sealed with crystal clear tape (Charles Supper Company). The 4-μL sitting drop (1:1 ratio) was covered with silicon oil, while the well contained 300 μL precipitant solution. Crystals were obtained in 10% PEG 8000, 100 mM MES pH 5.5 and 200 mM zinc acetate. Improved crystal quality was achieved by additive screens (Hampton Research). The best diffracted crystals were optimized in a precipitant composed of 10% PEG 8000, 0.1 M MES at pH 5.5, 0.2 M zinc acetate, 3% ethylene glycol, 3% glycerol, 10 mM cadmium chloride hydrate, 4% v/v polypropylene glycol P 400, and 3% v/v 2-propanol. Crystals formed after 3 to 5 days at 20°C and reached full size after ~20 days. Individual crystals were harvested using a MicroLoop LD 100 μm– 200 μm (MiTeGen) and flash frozen in liquid nitrogen without additional cryoprotectant.

### X-ray data collection and analysis

X-ray diffraction experiments were performed on beamline 23-ID-D of the Advanced Photon Source (Lemont, Illinois). Data were collected at 100 K using a 1.03348 Å, 19 μm x 16 μm x-ray beam that was attenuated by a factor of five. Reflections were collected using the vector (helical) method created at APS [68] over a total of 180° using an increment of 0.5° and 0.5 s per frame. The detector was a Pilatus3 6M and operated in a continuous, shutterless data collection mode at a distance of 350 mm. Diffraction data from a single large crystal was indexed and merged using the GMCAproc pipeline [69], which includes wrappers using XDS [70], POINTLESS [71], AIMLESS [72], and TRUNCATE [73]. The structure was solved by iterative molecular replacement with phenix.phaser [74] using the RBD2 from PDB ID 7bz5 [75] and the Fab from PDB ID 7beh [76]. When placing the Fab, the variable regions of the combined heavy and light chains were placed first, followed by the constant regions of the heavy and light chains. The model was manually built in Coot [77] and iterative refinement was performed using the phenix.refine routine of Phenix [78]. The X-ray data collection and refinement statistics are summarized in S2 Table. Figures were prepared using PyMOL (Schrodinger, LLC. Version 2.4.1).

### Antibody nebulization by a mesh-nebulizer

Macaques were anaesthetized after premedication with atropine (0.04 mg/kg) using ketamine (7 mg/kg, Imalgene 1000, Merial) and medetomidine (0.07 mg/kg, Domitor, Vétoquinol). Animals were awakened using atipamezol (0.2 mg/kg).

Macaques were treated with the antibody solution using an Aeroneb Solo mesh Nebulizer (MMAD approximately 4 μm) adapted to a facemask. A single Neb. of 25 mg/kg of antibody solution (25 mg/mL) was performed using a beaded paediatric facemask (Intersurgical, 15M, size 1) covering the mouth and nose. The macaques were placed in a dorsal recumbent position at 30° in a support cushion in a BSL3 biological safety cabinet. Antibody administration was performed during spontaneous breathing without oxygen supply. Oximetry, as well as the respiratory and heart rates, were monitored during administration until complete recovery.

### Estimation of ICO-hu104 deposited dose by [^18^F]-FDG PET/CT imaging extrapolation

In order to estimate the inhaled dose of ICO-hu104 we first characterized the particle size distribution of the antibody solution nebulized with three different commercial Aeroneb solo. The particle distribution was characterized by cascade impaction with a Dekati Low Pressure Impactor (DLPI+). In parallel we also characterized the particle distribution of a [^18^F]-FDG radioactive solution nebulized by the Aeroneb solo with the exact same device (Aeroneb connected with Y tube) as for animal treatment. DLPI+ operating at 40Mbar at ambient temperature. After nebulization, each plate of the impactor was rinsed with 4 mL of NaCl 0,9%. The radioactivity was counted, and the mass distribution calculated for each plate. The ICO-hu104 concentration in collected fractions was determined by ELISA assay as previously described and the mass deducted to plot the aerosol distribution. The antibody concentration in distribution fractions were estimated based on the concentration curve from the reference antibody using GraphPad Prism 9.2.0 software. The [^18^F]-FDG concentration was determined by gamma counting (Hidex AMG, LabLogic). Data were expressed as the percentage of total drug (or radioactivity) deposited on all stages of the impactor and are represented by the mean ± standard deviation (n = 3 nebulizations for each 2 compounds).

We use Positron Emission Tomography coupled to Computed Tomography (PET/CT) imaging (Vereos PET/CT, Philips Healthcare) to measure the total [^18^F]-FDG substance deposited for upper and lower respiratory tract just after nebulization. We then calculated the nebulizer device performances by measuring the radiotracer deposition pattern in the respiratory tract of six cynomolgus macaques weighing from 3,6 to 6,2 kg using LifeX software. The means and S.D. percentage of the initial dose (inside the nebulizer tank). Then we hypostatize that ICO-hu104 distribution is similar to the radiotracer one in the animal respiratory tract.

### Antibody efficacy study

Six female and four male cynomolgus macaques aged 2.5 to 4 years were randomly assigned to the control (n = 2) or treated groups to evaluate the efficacy of the ICO-hu104 antibody candidate, 10 control animals have been also added for analysis. The IV treated group (n  =  3) received one bolus dose of ICO-hu104 mAb (25 mg/kg) by the IV route in the saphenous vein and the nebulized treated group (n = 5) received a single treatment of antibody (25 mg/kg) one day after SARS-CoV-2 challenge, whereas extemporaneous control animals received only the diluent by Neb and historical control animals received nothing. All animals were exposed to a total dose of 10^5^ TCID50 of SARS-CoV-2 *Delta strain* (hCoV-19/USA/PHC658/2021, grown in Calu-3 cells) via the combination of intranasal and intratracheal routes (day 0), using atropine (0.04 mg/kg) for pre-medication and ketamine (5 mg/kg) with medetomidine (0.05 mg/kg) for anesthesia. Animals were observed daily. Clinical exams were performed at baseline, daily for one week, and then once or twice weekly on anaesthetized animals using ketamine (5 mg/kg) associated with medetomidine (0.05 mg/kg). Blood and nasopharyngeal and tracheal swabs were collected at the same timepoints. BAL of the whole lungs was performed using 50 ml sterile saline at baseline and at 3 and 10 d.p.i. after intratracheal intubation.

### Virus quantification in NHP samples

Upper respiratory (nasopharyngeal and tracheal) specimens were collected with swabs (Viral Transport Medium, CDC, DSR-052-01). Tracheal swabs were performed by insertion of the swab above the tip of the epiglottis into the upper trachea approximately 1.5 cm from the epiglottis. All specimens were stored between 2°C and 8°C until analysis by RT-qPCR with a plasmid standard concentration range containing an RdRp gene fragment that included the RdRp-IP4 RT-PCR target sequence. The limit of detection was estimated to be 2.67 log_10_ copies of SARS-CoV-2 gRNA per mL and the limit of quantification 3.67 log_10_ copies per mL. SARS-CoV-2 sgRNA levels were assessed by RT-qPCR targeting the E gene using previously described primers and probes [36,79]: leader-specific primer sgLeadSARSCoV2-F CGATCTCTTGTAGATCTGTTCTC, E-Sarbeco-R primer ATATTGCAGCAGTACGCACACA, and E-Sarbeco probe HEX579 ACACTAGCCATCCTTACTGCGCTTCG-BHQ1. The protocol describing the procedure for the detection of SARS-CoV-2 is available on the WHO website (https://www.who.int/docs/default-source/coronaviruse/real-time-rt-pcr-assays-for-the-detection-of-sars-cov-2-institut-pasteur-paris.pdf?sfvrsn=3662fcb6_2). The limit of detection was estimated to be 2.87 log_10_ copies of SARS-CoV-2 sgRNA per mL and the limit of quantification 3.87 log_10_ copies per mL.

### Measurement of ICO-hu104 antibody concentrations in Cynomolgus Macaque biological samples

The concentration of antibody ICO-hu104 in the biological samples was measured using an ELISA-based binding assay. The wells of Nunc MaxiSorp flat-bottom 96-well plates (Thermo Fischer Scientific, catalog number: 468667) were coated with purified SARS-CoV-2 Delta strain (B.1.617.2) trimeric S-protein (Icosagen Cell Factory, catalog number P-353-100) at 1 μg/mL in PBS overnight at 4°C and blocked with a PBS solution containing 1% BSA and 0.1% ProClin 300 for 1 h at RT. The samples (biological samples: heat-inactivated serum, BAL, and tracheal and nasopharyngeal swabs from Cynomolgus Macaques and purified antibody ICO-hu104 as interpretation standard) were diluted in assay buffer (1% BSA in PBS) and added to the plate by three-fold serial dilution in a final volume of 100 μL. Samples were incubated for 1 h at RT at 450 rpm. Then, goat anti-human IgG secondary antibody was added at a 1:10,000 dilution and the plates incubated for 30 min at RT at 450 rpm. Colorimetric development was performed using 3,3’,5,5’-tetramethylbenzidine VII substrate, the reaction stopped using 0.5 mol/L H_2_SO_4_, and the absorbance measured at 450 nm. The antibody concentration in biological samples was estimated based on the concentration curve from the reference antibody using GraphPad Prism 9.2.0 software.

### Antibody quantification

Cynomolgus macaque’s serum samples were screened for Spike-specific IgG against the SARS-CoV-2 Delta variant using the V-PLEX SARS-CoV-2 Panel 13 and Panel Spike 01 kits (IgG, MesoScale Discovery [MSD], Rockville, USA) according to the manufacturer’s instructions. The plates were blocked with 50 μL of blocker A solution (1% BSA in MilliQ water) for at least 30 min at room temperature. During blocking, heat-inactivated serum samples were diluted 1:500, 1:5,000, and 1:50,000 in a diluent buffer. Each plate contained duplicates of a seven-point calibration curve with serial dilution of a reference standard and a blank well. The plates were then washed three times with 150 μL of the MSD kit wash buffer, blotted dry, and 50 μL of the diluted serum samples were added to the plates. After 2h of incubation at room temperature, the plates were washed three times and 50 μL of SULFO-Tagged anti-human IgG antibody was added to each well and incubated at room temperature for at least 1 h. Plates were then washed three times and 150 μL of MSD GOLD Read Buffer B was added to each well. The plates were read immediately using a MESO QuickPlex SQ 120 machine. Electro-chemiluminescence (ECL) signal was recorded and the results were expressed as arbitrary units (AU)/mL.

### Plasma cytokine analysis

Cytokines were quantified in EDTA-treated plasma using NHP Milliplex kit (Millipore) and a Bioplex 200 analyzer (Bio-Rad), according to manufacturer’s instructions.

### Biochemistry

Biochemistry parameters including ALAT, ASAT, GammaGT, LDH, haptoglobin, phosphatase alkaline (ALP), cholesterol, urea, creatinine, glycemia and C-reactive protein were analyzed in Triton X-100 inactivated serum, using standard kits (Siemens) or canine kit (Randox) and an ADVIA1800 analyzer (Siemens).

### Statistical analysis

Statistical analyses were done using Kruskal-Wallis test followed by Mann-Whitney test. For PK/PD analysis, the area under the curve for viral load were calculated after log10 transformation of the number of RNA copies/mL.

## Supporting information

S1 Fig An antibody isolated from a convalescent patient shows neutralizing activity against SARS-CoV-2. **A.** SARS-CoV-2 virus neutralization assay in VERO E6 cells demonstrating minimal antibody concentrations with no detectable cytopathic effects in response to viral infection. Top neutralizing antibody clones selected for further in-depth characterization are color coded. **B.** ELISA-based EC50 measurement against trimeric Spike protein of antibodies isolated from convalescent patients. **C.** Binding affinities of the developed SARS-CoV-2 virus neutralizing antibodies towards trimeric-Spike (y-axis) and the monomeric S1-domain (x-axis).
(TIFF)

S2 Fig Antibody ICO-hu23 reduces the viral load in Syrian golden hamsters in both prophylactic and therapeutic settings. **A.** Schematic overview of the study protocol of the Syrian golden hamster SARS-CoV-2 model in a prophylactic and therapeutic setting. Animals (n = 32 per study group) were infected intranasally at day 0, with antibody administration either 24 h before or after infection. **B., C.** SARS-CoV-2 RNA viral load levels relative to those of γ–actin in the RdRp-IP4 assay. All tested antibody concentrations resulted in a significant reduction of viral load in the prophylactic setting (B). In the therapeutic study setting (C), the 5 mg/kg and 0.75 mg/kg administered doses resulted in a significant reduction of viral load relative to the control group.
(TIFF)

S3 Fig ICO-hu23 antibody loses neutralizing functionality towards certain VoC’s in a pseudovirus assay. Pseudovirus neutralization assay of ICO-hu23 antibody with the Wuhan, Alpha, Beta, Delta, Omicron, and Omicron BA.2 VoCs.
(TIFF)

S4 Fig ELISA assay of antibody clones isolated from rabbit splenocytes capable of blocking the interaction between ACE2 and Spike trimers of Beta VoC. The relative OD450 is calculated based on negative control and sample OD values.
(TIFF)

S5 Fig Antibody ICO-hu104 shows subnanomolar affinities against SARS-CoV-2 VoCs. **A.** Biolayer interferometry assay (BLI) for measuring the ICO-hu104 antibody binding kinetics to trimeric Spike proteins of the original SARS-CoV-2 Wuhan strain and five VoCs. Antibody was loaded onto Protein A biosensors and the association with trimeric Spike protein was measured for 5 min (600 s). Trimeric Spike proteins were used as an analyte at five different concentrations and the data was analyzed using the 1:1 Global Fit model. **B.** kON, kOFF, and kD values characterizing the BLI measurements.
(TIFF)

S6 Fig Serology against SARS-CoV-2 Delta. Spike-binding IgG concentration (arbitrary units [AU]/mL). Serum from NHPs included in the study were evaluated for IgG response against Spike Delta before challenge (BL) and at week 4 (W4) post challenge. The dotted line represents the limit of positivity previously determined with cynomolgus macaques.
(TIFF)

S7 Fig Deposited dose calculation by nuclear imaging after Aeroneb administration. **A.** Size distribution assessment of aerosolized droplets by cascade impaction for both the monoclonal antibody ICO-hu104 (blue) and [^18^F]-FDG (orange). Data are represented as mean +/- S.D. (n = 3 measurements for each molecule). **B.** Representative images of PET/CT imaging in cynomolgus macaque after [^18^F]-FDG nebulization *in vivo*. **C.** percentages of [^18^F]-FDG deposition in the whole respiratory tract, the upper respiratory tract, and the lungs. Data are represented as median +/- S.D. (n = 6 animals). Below each panel is calculated the associated estimation of antibody dose for each compartment.
(TIFF)

S8 Fig Antibody ICO-hu104 is capable of reducing viral load in tracheal fluids through nebulization and intravenous administration. **A.** Genomic (g)RNA in tracheal fluids is expressed as viral copies per ml. Individual values are plotted by group. Median values are represented by the thick line. The dotted line represents the LOQ = 4760 for gRNA. **B.** The area under the curve for data from days 0 to 14 (AUC_0-14_) of the gRNA VL is shown for each individual by group. Grey circles represent the historical controls and the black circles the extemporaneous controls, red squares the intravenous treatment group, and blue triangles the nebulization treatment group. **C.** Subgenomic (sg)RNA determined by PCR in tracheal fluids. Individual values are plotted by group. Median values are represented by the thick line. The dotted line represents the LOQ = 7490 for sgRNA. Kruskal-Wallis test p = 0.2845. CTRL: control group, IV: intravenous treatment, Neb: nebulization treatment, LOQ: limit of quantification.
(TIFF)

S9 Fig Cytokines and chemokines in the plasma of SARS-CoV-2-infected cynomolgus macaques. Different cytokines and chemokines expression were measured at baseline (BL) and at day 2 post challenge (D2) corresponding to the peak of inflammation: A: IL15, B: IL1RA, C: MCP1. The line represents the median value and the asterisk indicates a significant difference in IL15 and IL-1RA concentrations at 2 d.p.i. between the control group and TT IV. group (p = 0.0245 and p = 0.0070 respectively). Simultaneous controls are in black, historical controls were included for the analysis and are represented in grey. Statistical significance was determined using a Mann–Whitney test.
(TIFF)

S10 Fig Biochemistry analysis in serum during the first week of infection. **A-K:** ALAT, ASAT, Gamma GT, phosphatase alkaline (ALP), LDH, glycemia, cholesterol, urea, creatinine, and CRP (Protein C-reactive) were analyzed in the sera of the NHP. In black: controls, Red: IV. TT and in Blue: Neb. TT.
(TIFF)

S11 Fig Cryo-EM reconstruction of the SARS-CoV-2 Spike trimer–Fab ICO-hu23 complex. **A.** Side and top views of the cryo-EM structure of the SARS-CoV-2 Spike trimer in the closed state with three Fab ICO-hu23 fragments bound. Each Spike protomer is shown in pink, yellow, and grey, whereas the Fab ICO-hu23 light chain and heavy chain variable domains are shown in cyan and dark green, respectively. **B.** Close-up views of the binding of the Fab ICO-hu23 variable domains to the Spike protein RBD. The same color scheme as in A is used for the Fab and RBD, with the CDR regions of the Fab and Phe-486 residue of RBD highlighted in different colors and labelled appropriately. Representative electron micrograph (**C**) and 2D class averages (**D**) of the SARS-CoV-2 Spike trimer in complex with Fab ICO-hu23. Fourier shell correlation (FSC) curve based on the (**E**) half-maps (FSC = 0.143) and (**F**) model-map (FSC = 0.5). **G.** Refined cryo-EM map colored by local resolution. **H.** A model is merged with a density map, with the Spike trimer in the closed state with three Fab ICO-hu23 fragments bound to the RBD. Each Spike protomer is shown in pink, yellow, and dark grey, whereas the Fab ICO-hu23 light chain and heavy chain variable domains are shown in cyan and dark green.
(TIFF)

S12 Fig X-ray crystal structure of a SARS-CoV-2 RBD–Fab ICO-hu23 complex. **A.** The asymmetric unit contains two molecules of RBD (cyan) and two molecules of Fab ICO-hu23 (heavy chain (VH), magenta; light chain (VL), yellow). **B.** At the RBD–Fab ICO-hu23 interface, Glu-484 of the RBD forms extensive polar interactions with three residues of the Fab ICO-hu23 VH. **C** Phe-486 of the RBD is surrounded by five aromatic residues, three of VL and two of VH. 2Fo-Fc electron density maps are contoured at 1.5 σ (blue mesh).
(TIFF)

S13 Fig Alignment of SARS-CoV-2 Wuhan strain Spike proteins. All mutations are highlighted against reference genome extracted from NCBI database: NC_045512.2.
(TIFF)

S1 Table Cryo-EM data collection, refinement, and validation statistics. (PDF)

S2 Table Crystallography data collection and refinement statistics. (PDF)

S3 Table Interfacial residues between RBD and Fab ICO-hu23 identified by PISA [42]. The interface interactions are characterized as van der Waals (vdW, 4.0 Å cut-off) or hydrogen bonds (HB, 3.8 Å cut-off).
(PDF)

S1 Data Minimal data file. (XLSX)
