# Supplementary figures and images for: Broadly neutralizing humanized SARS-CoV-2 antibody binds to a conserved epitope on Spike and provides antiviral protection through inhalation-based delivery in non-human primates
Source: PLoS Pathog. 2023 Aug 2;19(8):e1011532. doi: 10.1371/journal.ppat.1011532 (PMC10395824; doi:10.1371/journal.ppat.1011532)

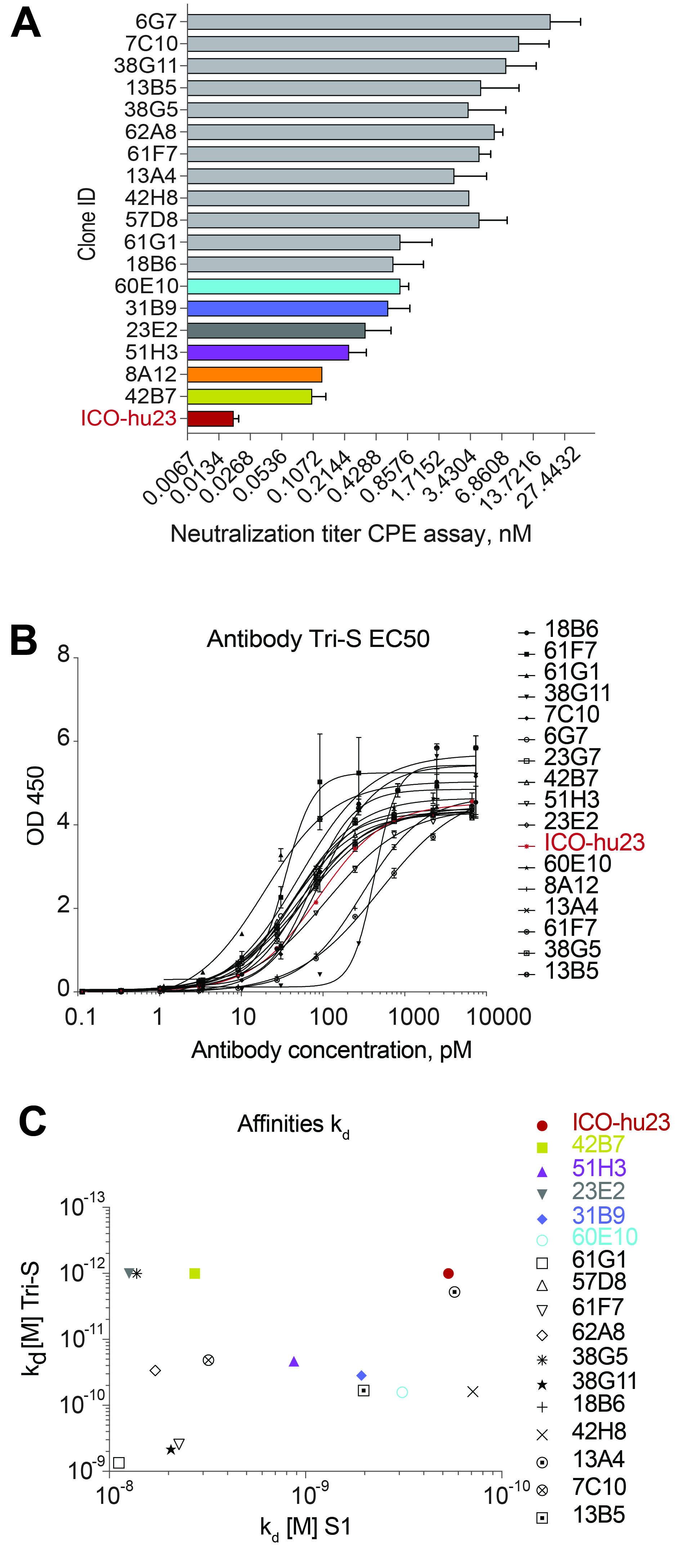

Supplement: S1 Fig — A. SARS-CoV-2 virus neutralization assay in VERO E6 cells demonstrating minimal antibody concentrations with no detectable cytopathic effects in response to viral infection. Top neutralizing antibody clones selected for further in-depth characterization are color coded. B. ELISA-based EC50 measurement against trimeric Spike protein of antibodies isolated from convalescent patients. C. Binding affinities of the developed SARS-CoV-2 virus neutralizing antibodies towards trimeric-Spike (y-axis) and the monomeric S1-domain (x-axis). (TIFF) [file ppat.1011532.s001.tiff]

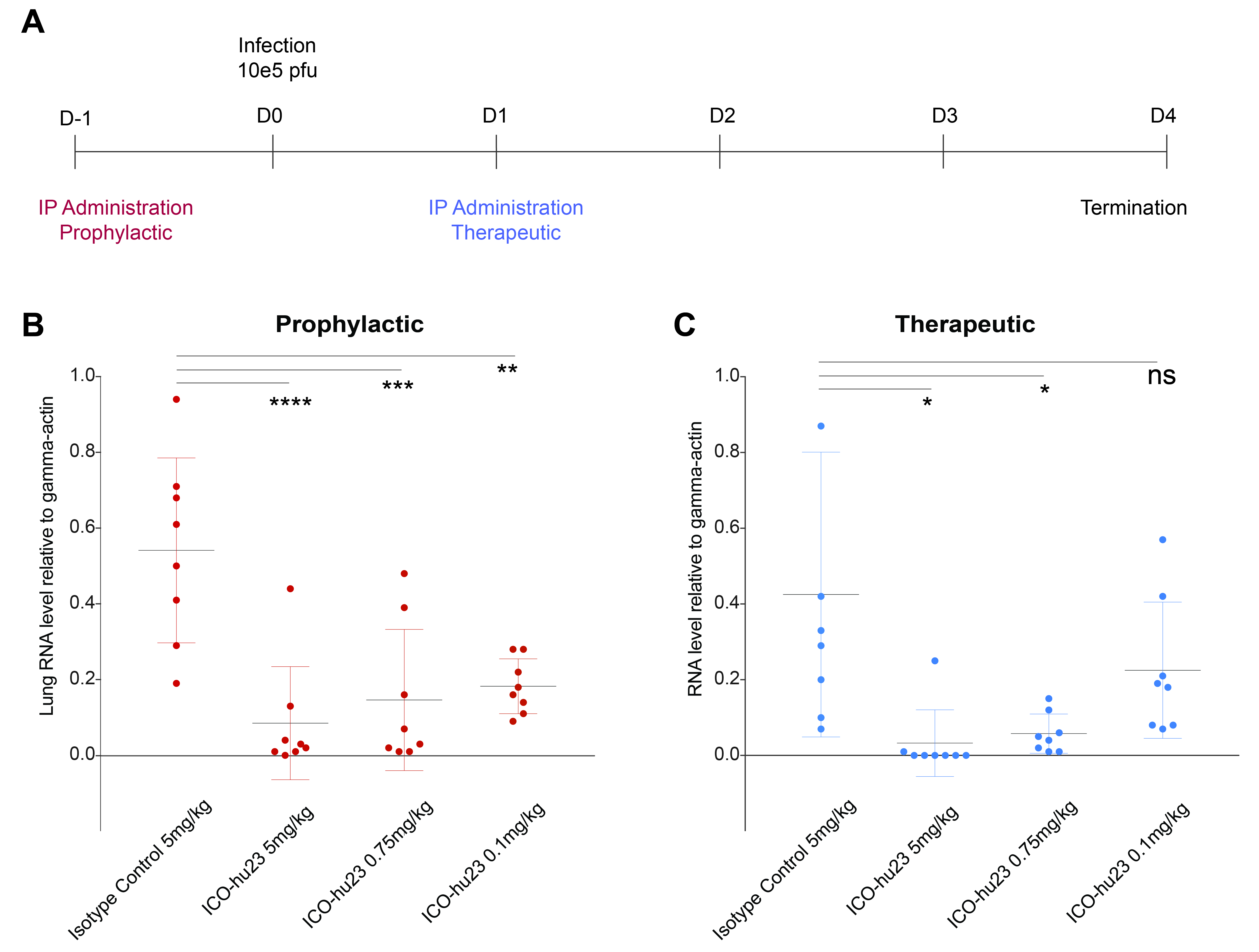

Supplement: S2 Fig — A. Schematic overview of the study protocol of the Syrian golden hamster SARS-CoV-2 model in a prophylactic and therapeutic setting. Animals (n = 32 per study group) were infected intranasally at day 0, with antibody administration either 24 h before or after infection. B., C. SARS-CoV-2 RNA viral load levels relative to those of γ–actin in the RdRp-IP4 assay. All tested antibody concentrations resulted in a significant reduction of viral load in the prophylactic setting (B). In the therapeutic study setting (C), the 5 mg/kg and 0.75 mg/kg administered doses resulted in a significant reduction of viral load relative to the control group. (TIFF) [file ppat.1011532.s002.tiff]

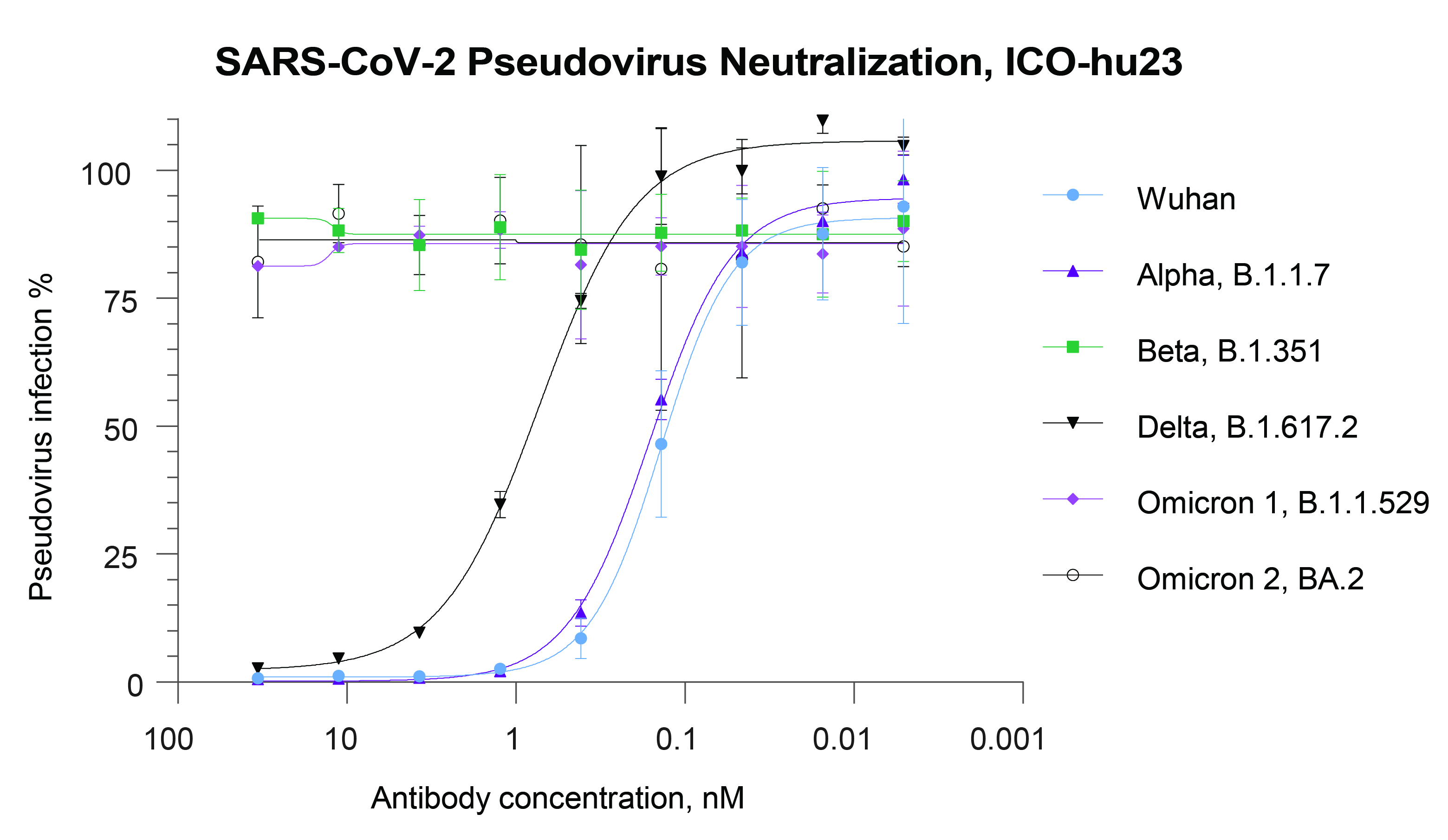

Supplement: S3 Fig — Pseudovirus neutralization assay of ICO-hu23 antibody with the Wuhan, Alpha, Beta, Delta, Omicron, and Omicron BA.2 VoCs. (TIFF) [file ppat.1011532.s003.tiff]

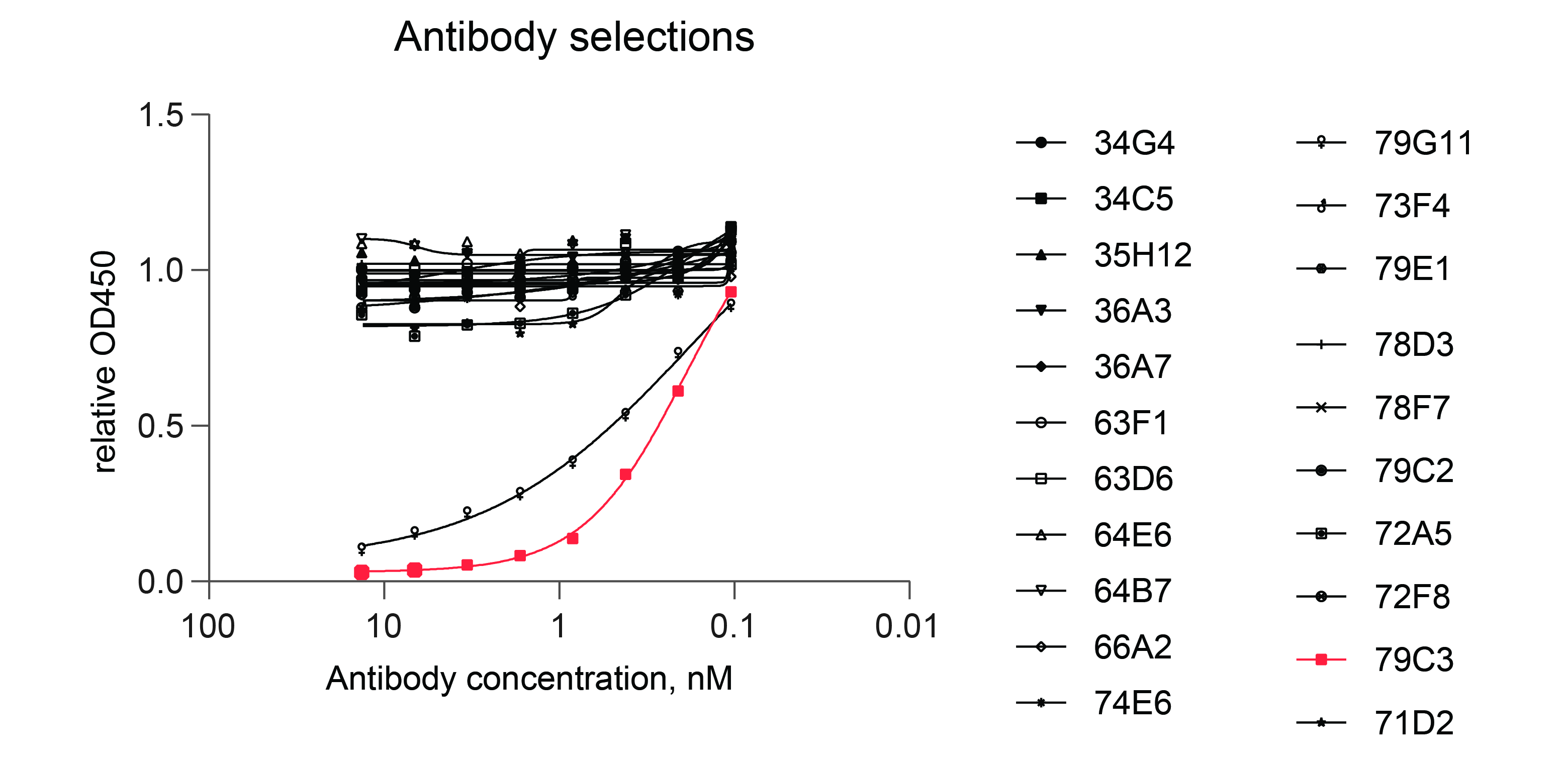

Supplement: S4 Fig — The relative OD450 is calculated based on negative control and sample OD values. (TIFF) [file ppat.1011532.s004.tiff]

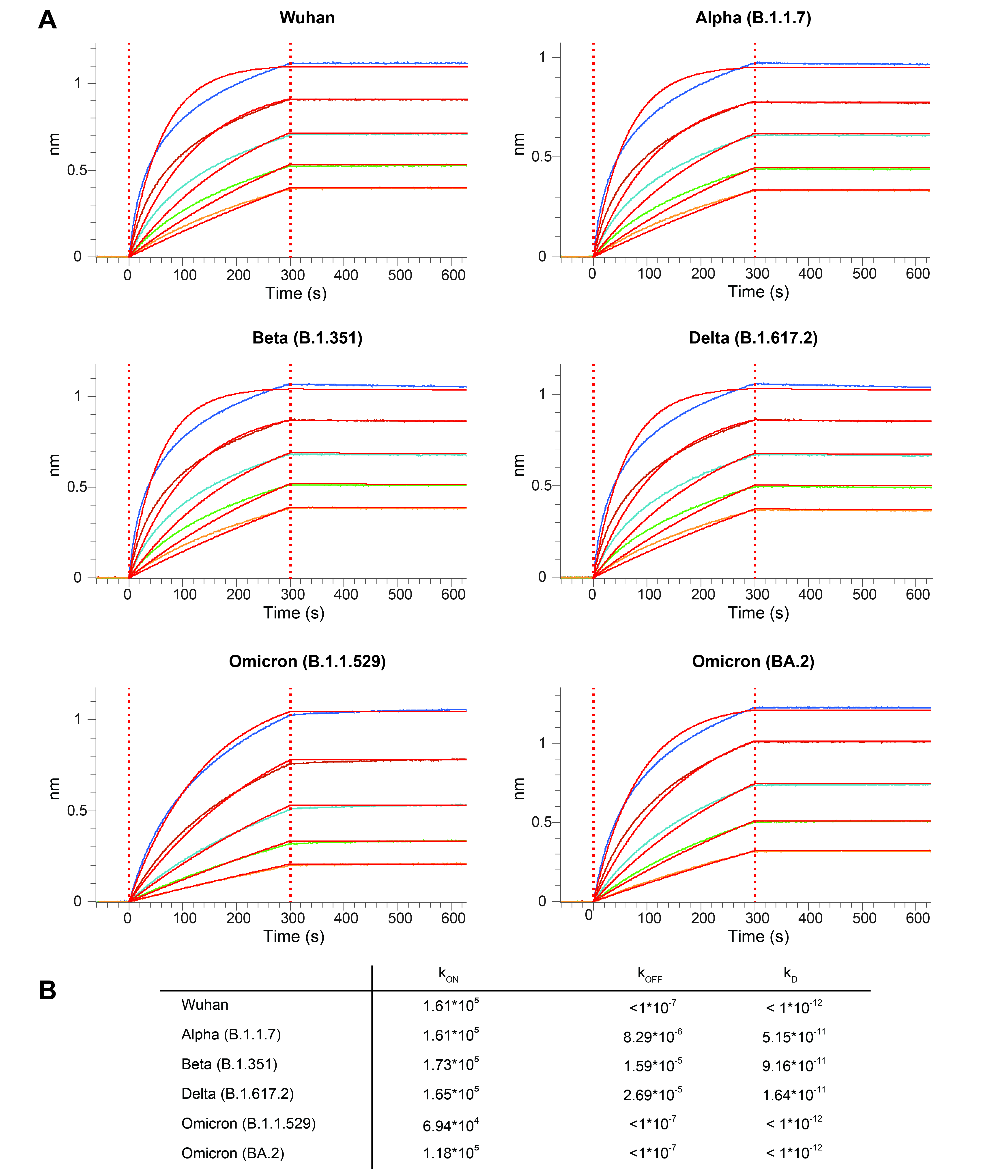

Supplement: S5 Fig — A. Biolayer interferometry assay (BLI) for measuring the ICO-hu104 antibody binding kinetics to trimeric Spike proteins of the original SARS-CoV-2 Wuhan strain and five VoCs. Antibody was loaded onto Protein A biosensors and the association with trimeric Spike protein was measured for 5 min (600 s). Trimeric Spike proteins were used as an analyte at five different concentrations and the data was analyzed using the 1:1 Global Fit model. B. kON, kOFF, and kD values characterizing the BLI measurements. (TIFF) [file ppat.1011532.s005.tiff]

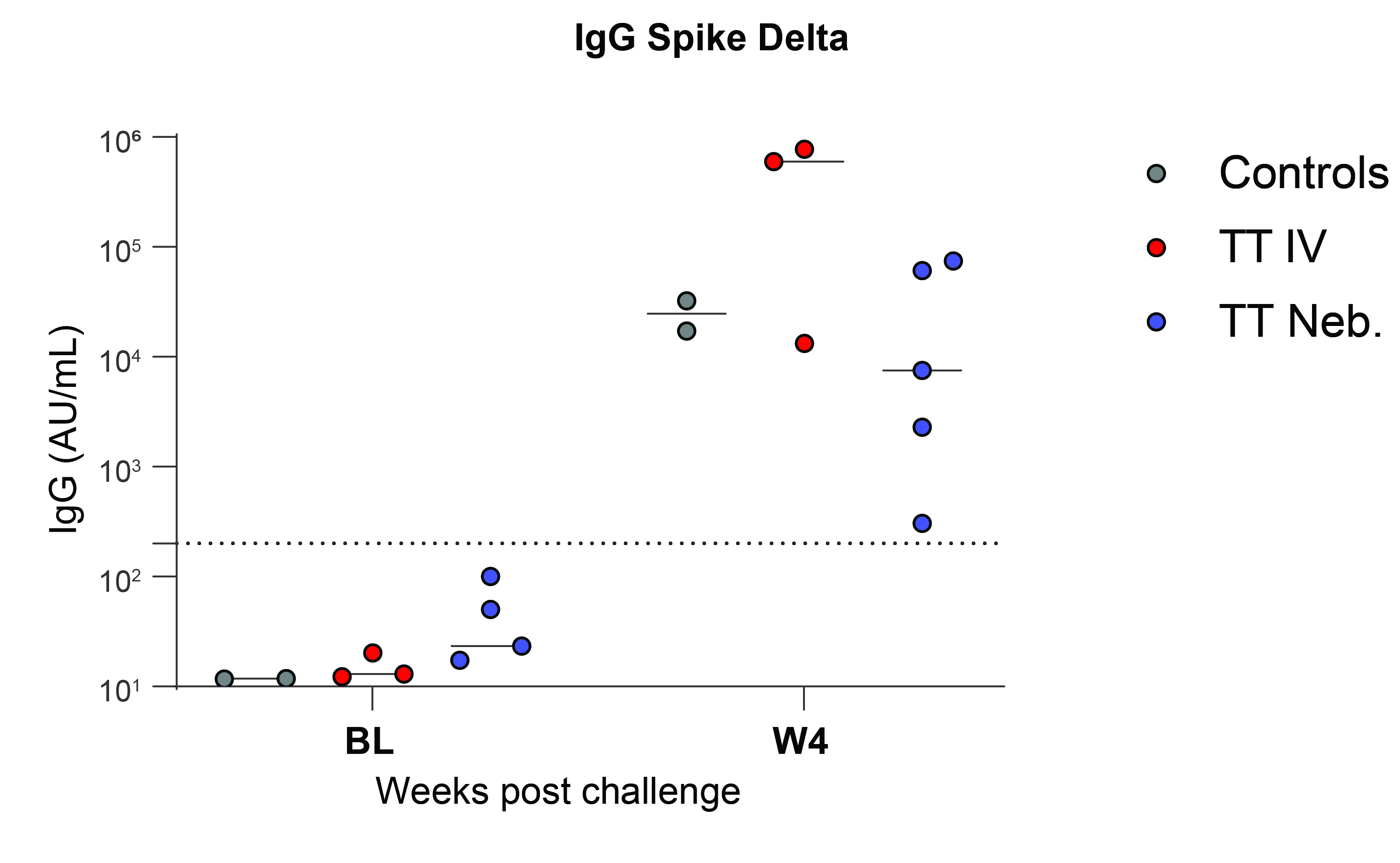

Supplement: S6 Fig — Spike-binding IgG concentration (arbitrary units [AU]/mL). Serum from NHPs included in the study were evaluated for IgG response against Spike Delta before challenge (BL) and at week 4 (W4) post challenge. The dotted line represents the limit of positivity previously determined with cynomolgus macaques. (TIFF) [file ppat.1011532.s006.tiff]

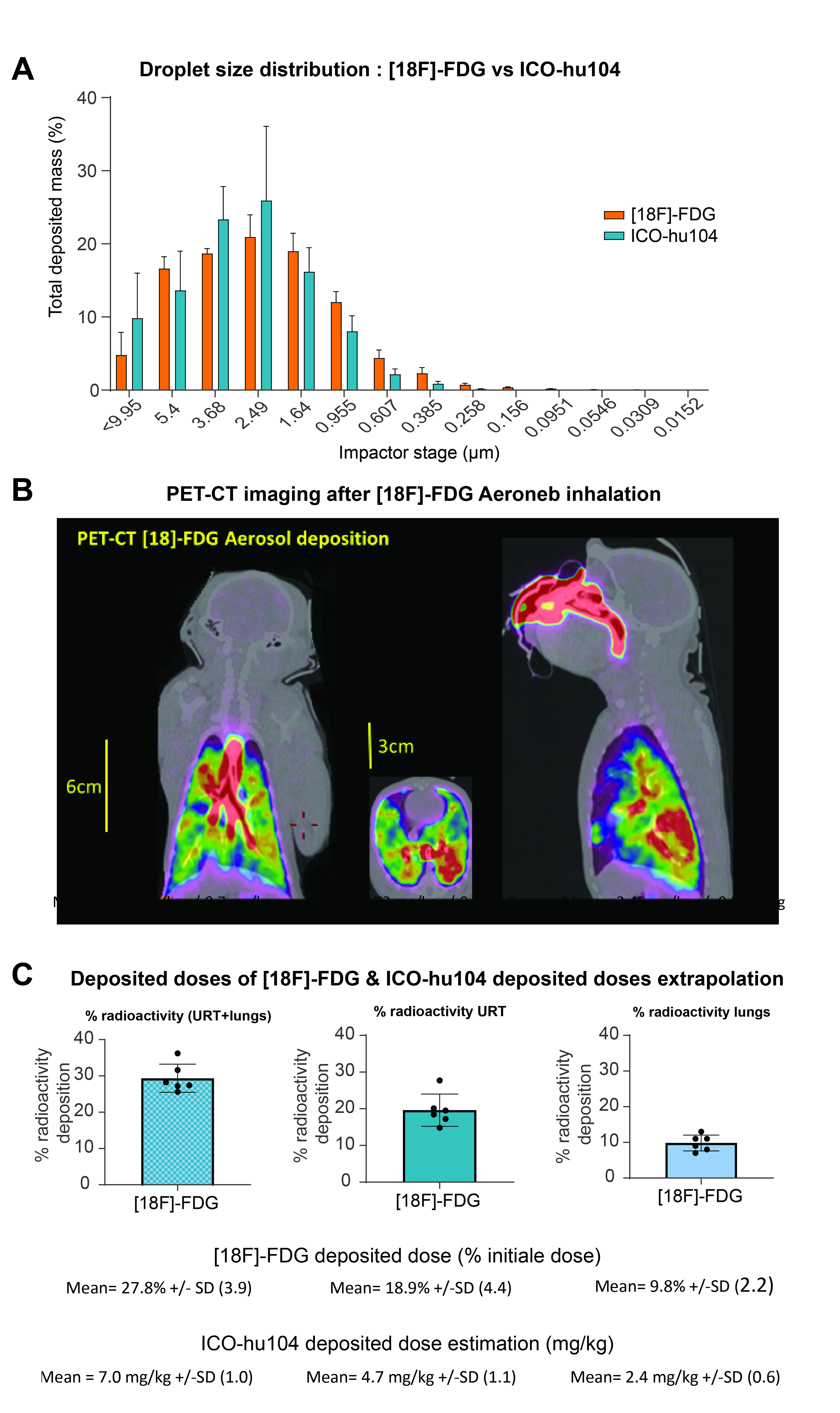

Supplement: S7 Fig — A. Size distribution assessment of aerosolized droplets by cascade impaction for both the monoclonal antibody ICO-hu104 (blue) and [18F]-FDG (orange). Data are represented as mean +/- S.D. (n = 3 measurements for each molecule). B. Representative images of PET/CT imaging in cynomolgus macaque after [18F]-FDG nebulization in vivo. C. percentages of [18F]-FDG deposition in the whole respiratory tract, the upper respiratory tract, and the lungs. Data are represented as median +/- S.D. (n = 6 animals). Below each panel is calculated the associated estimation of antibody dose for each compartment. (TIFF) [file ppat.1011532.s007.tiff]

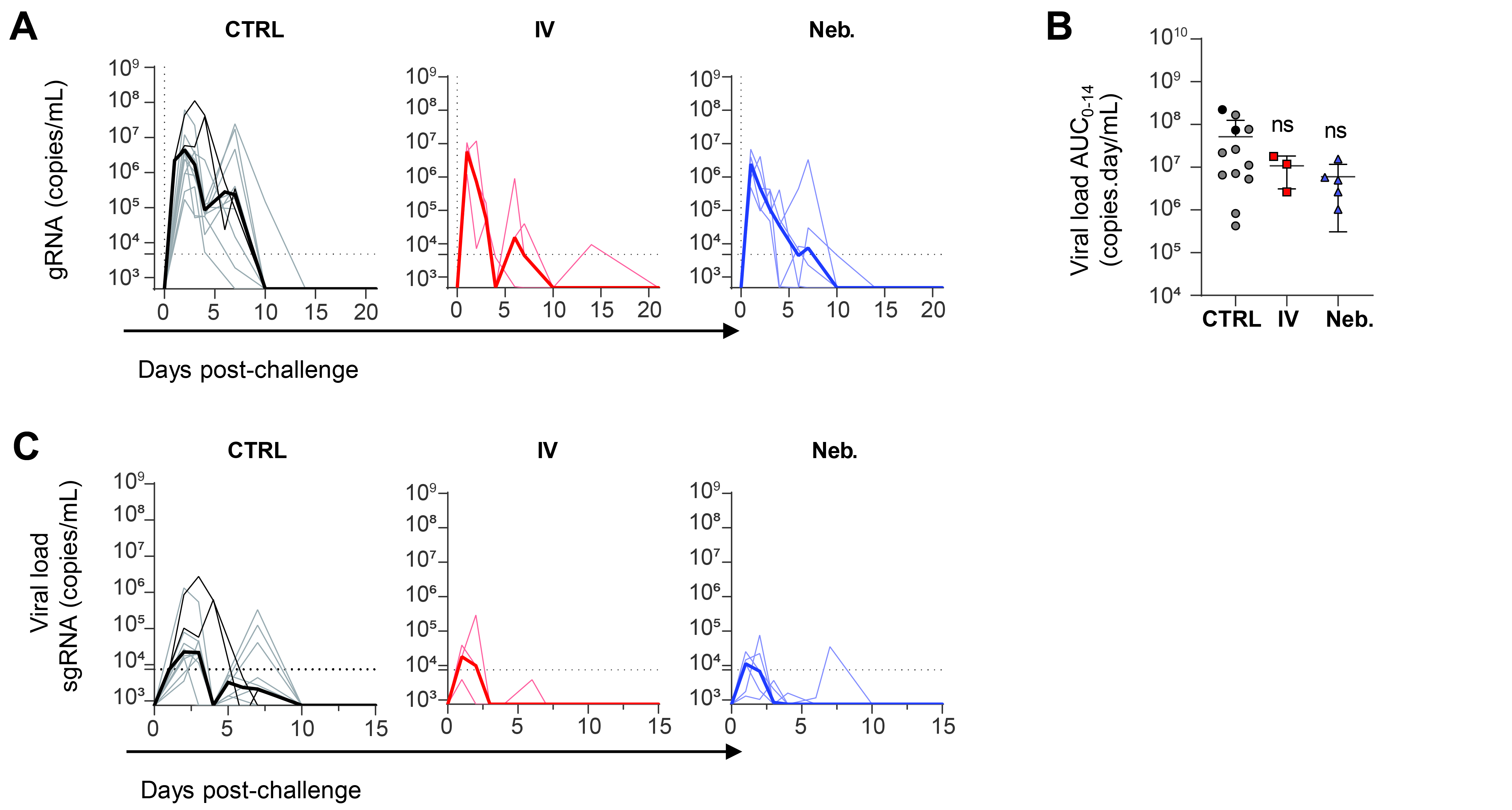

Supplement: S8 Fig — A. Genomic (g)RNA in tracheal fluids is expressed as viral copies per ml. Individual values are plotted by group. Median values are represented by the thick line. The dotted line represents the LOQ = 4760 for gRNA. B. The area under the curve for data from days 0 to 14 (AUC0-14) of the gRNA VL is shown for each individual by group. Grey circles represent the historical controls and the black circles the extemporaneous controls, red squares the intravenous treatment group, and blue triangles the nebulization treatment group. C. Subgenomic (sg)RNA determined by PCR in tracheal fluids. Individual values are plotted by group. Median values are represented by the thick line. The dotted line represents the LOQ = 7490 for sgRNA. Kruskal-Wallis test p = 0.2845. CTRL: control group, IV: intravenous treatment, Neb: nebulization treatment, LOQ: limit of quantification. (TIFF) [file ppat.1011532.s008.tiff]

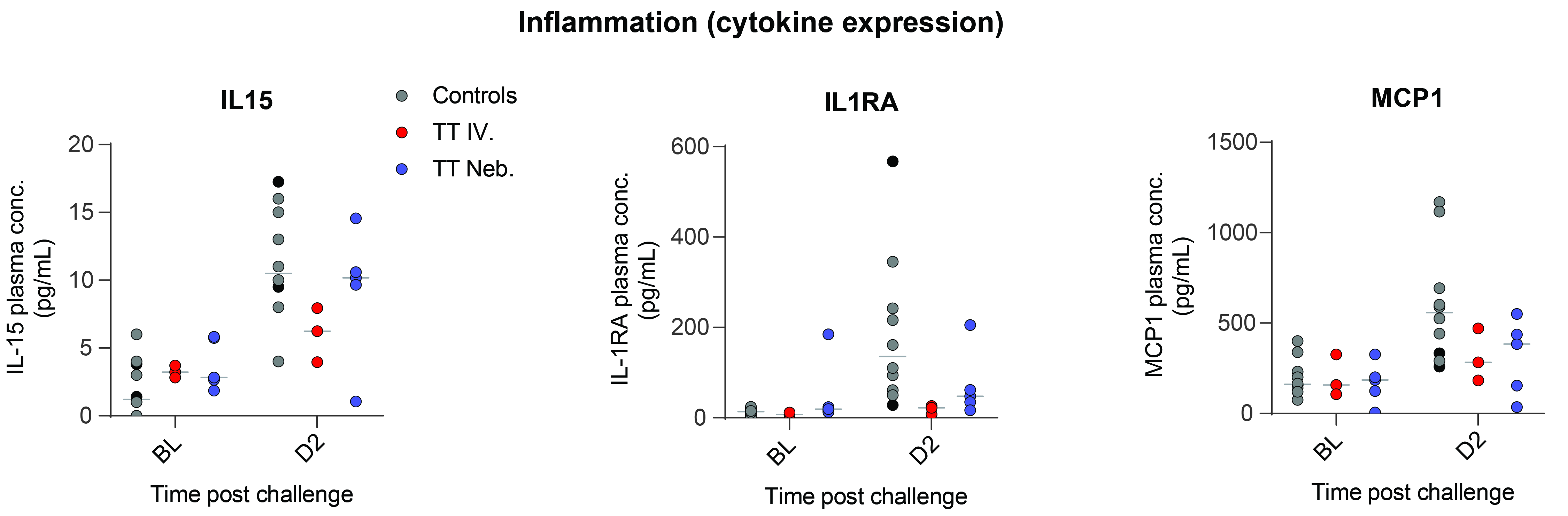

Supplement: S9 Fig — Different cytokines and chemokines expression were measured at baseline (BL) and at day 2 post challenge (D2) corresponding to the peak of inflammation: A: IL15, B: IL1RA, C: MCP1. The line represents the median value and the asterisk indicates a significant difference in IL15 and IL-1RA concentrations at 2 d.p.i. between the control group and TT IV. group (p = 0.0245 and p = 0.0070 respectively). Simultaneous controls are in black, historical controls were included for the analysis and are represented in grey. Statistical significance was determined using a Mann–Whitney test. (TIFF) [file ppat.1011532.s009.tiff]

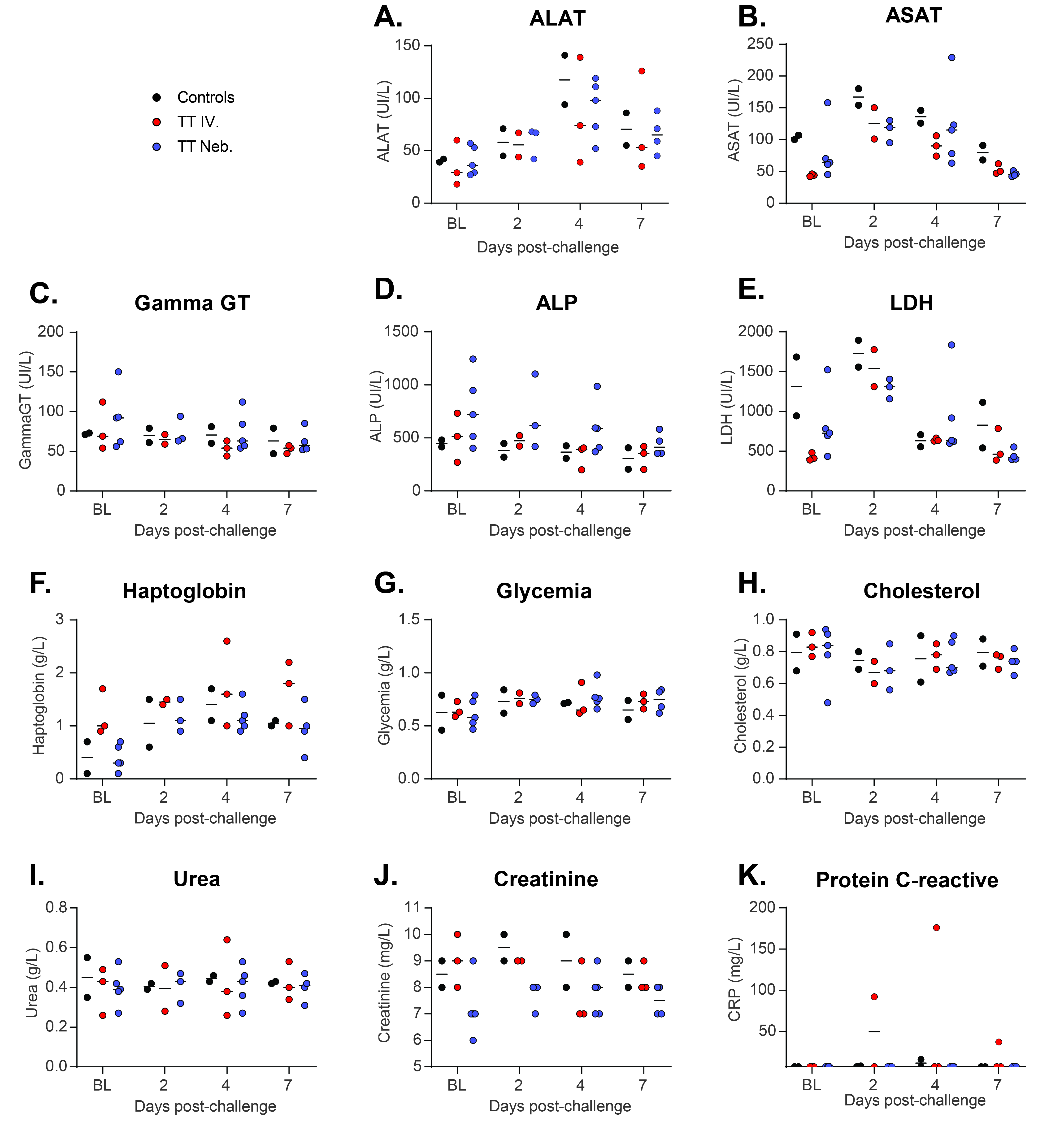

Supplement: S10 Fig — A-K: ALAT, ASAT, Gamma GT, phosphatase alkaline (ALP), LDH, glycemia, cholesterol, urea, creatinine, and CRP (Protein C-reactive) were analyzed in the sera of the NHP. In black: controls, Red: IV. TT and in Blue: Neb. TT. (TIFF) [file ppat.1011532.s010.tiff]

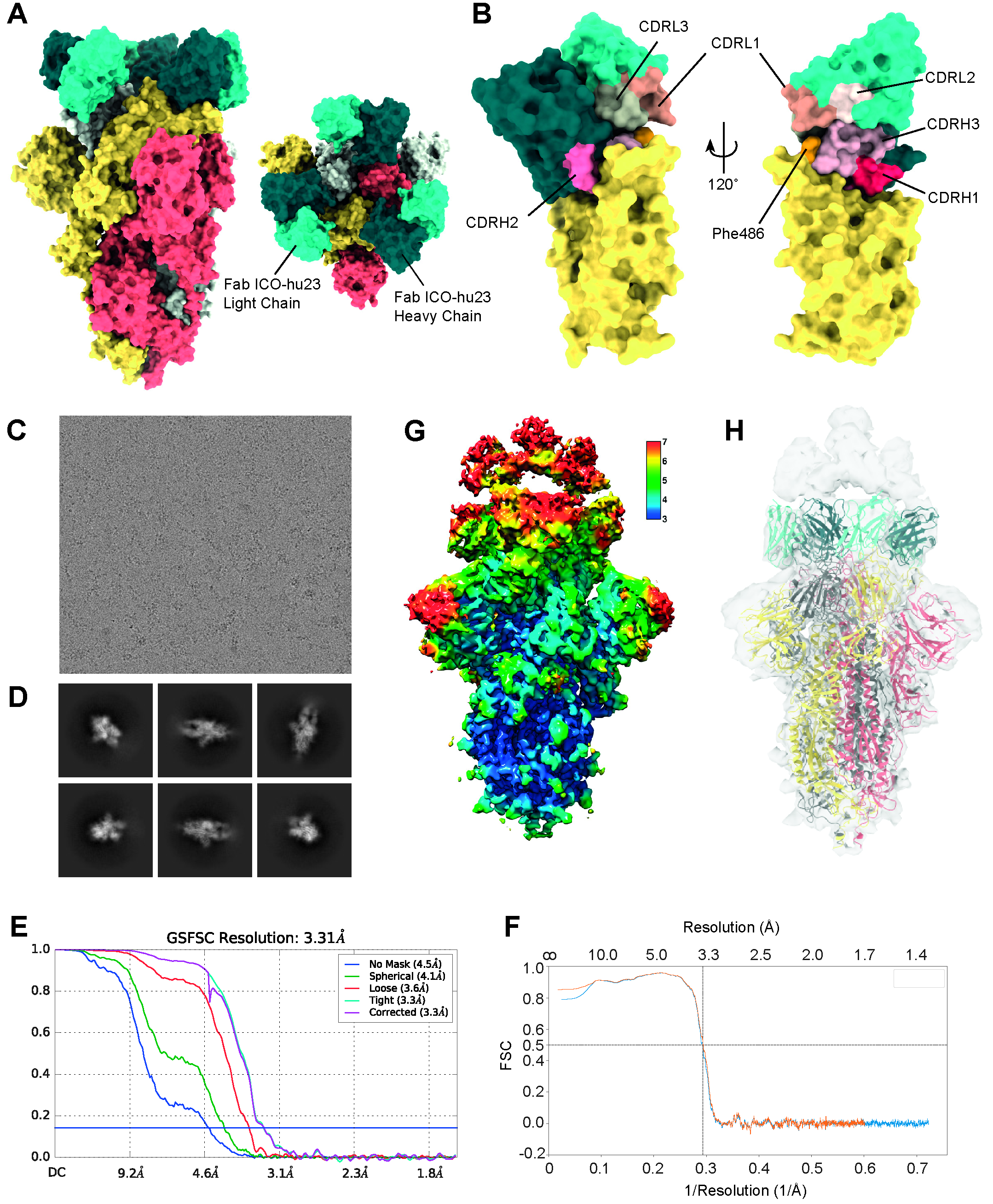

Supplement: S11 Fig — A. Side and top views of the cryo-EM structure of the SARS-CoV-2 Spike trimer in the closed state with three Fab ICO-hu23 fragments bound. Each Spike protomer is shown in pink, yellow, and grey, whereas the Fab ICO-hu23 light chain and heavy chain variable domains are shown in cyan and dark green, respectively. B. Close-up views of the binding of the Fab ICO-hu23 variable domains to the Spike protein RBD. The same color scheme as in A is used for the Fab and RBD, with the CDR regions of the Fab and Phe-486 residue of RBD highlighted in different colors and labelled appropriately. Representative electron micrograph (C) and 2D class averages (D) of the SARS-CoV-2 Spike trimer in complex with Fab ICO-hu23. Fourier shell correlation (FSC) curve based on the (E) half-maps (FSC = 0.143) and (F) model-map (FSC = 0.5). G. Refined cryo-EM map colored by local resolution. H. A model is merged with a density map, with the Spike trimer in the closed state with three Fab ICO-hu23 fragments bound to the RBD. Each Spike protomer is shown in pink, yellow, and dark grey, whereas the Fab ICO-hu23 light chain and heavy chain variable domains are shown in cyan and dark green. (TIFF) [file ppat.1011532.s011.tiff]

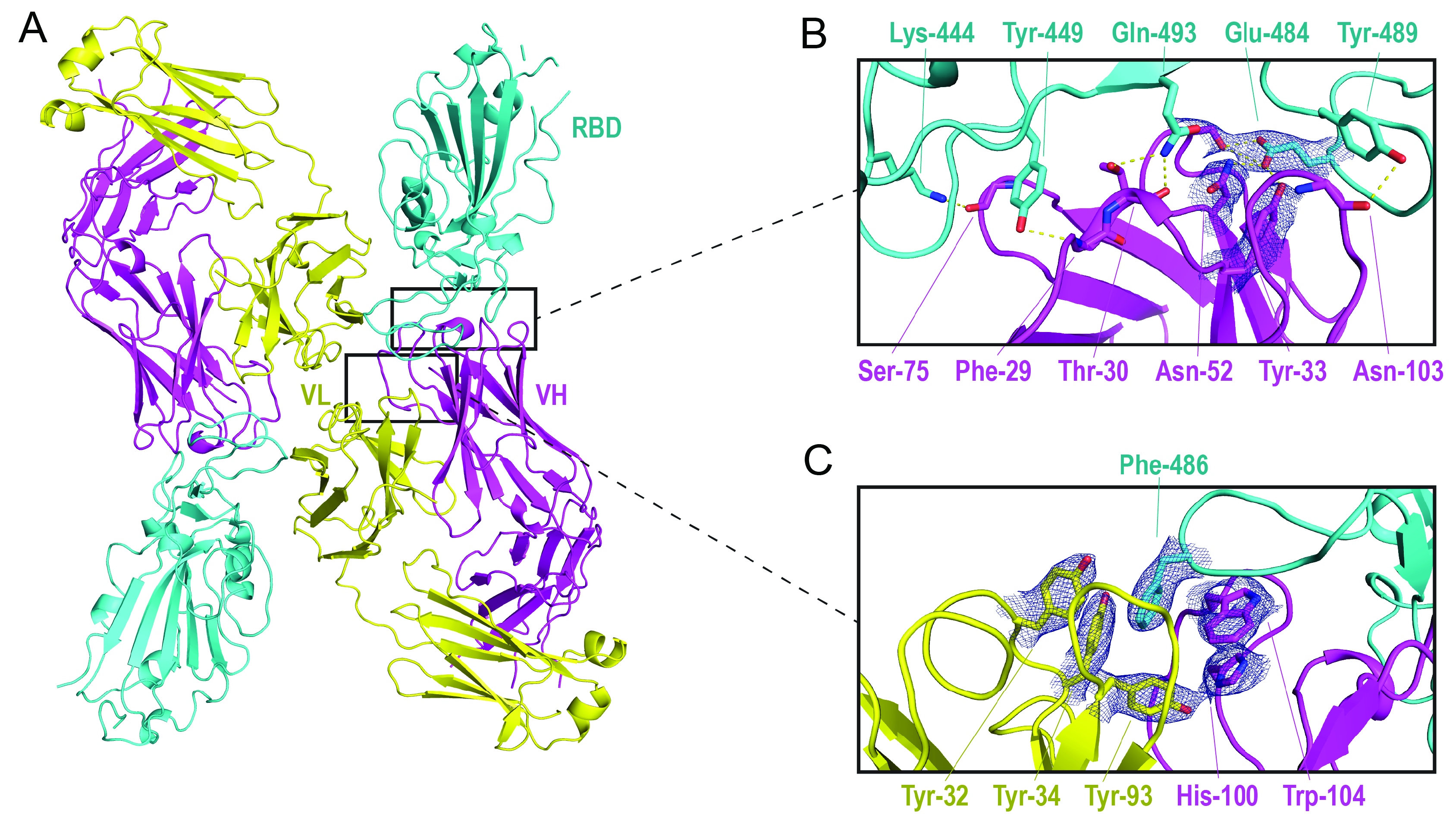

Supplement: S12 Fig — A. The asymmetric unit contains two molecules of RBD (cyan) and two molecules of Fab ICO-hu23 (heavy chain (VH), magenta; light chain (VL), yellow). B. At the RBD–Fab ICO-hu23 interface, Glu-484 of the RBD forms extensive polar interactions with three residues of the Fab ICO-hu23 VH. C Phe-486 of the RBD is surrounded by five aromatic residues, three of VL and two of VH. 2Fo-Fc electron density maps are contoured at 1.5 σ (blue mesh). (TIFF) [file ppat.1011532.s012.tiff]

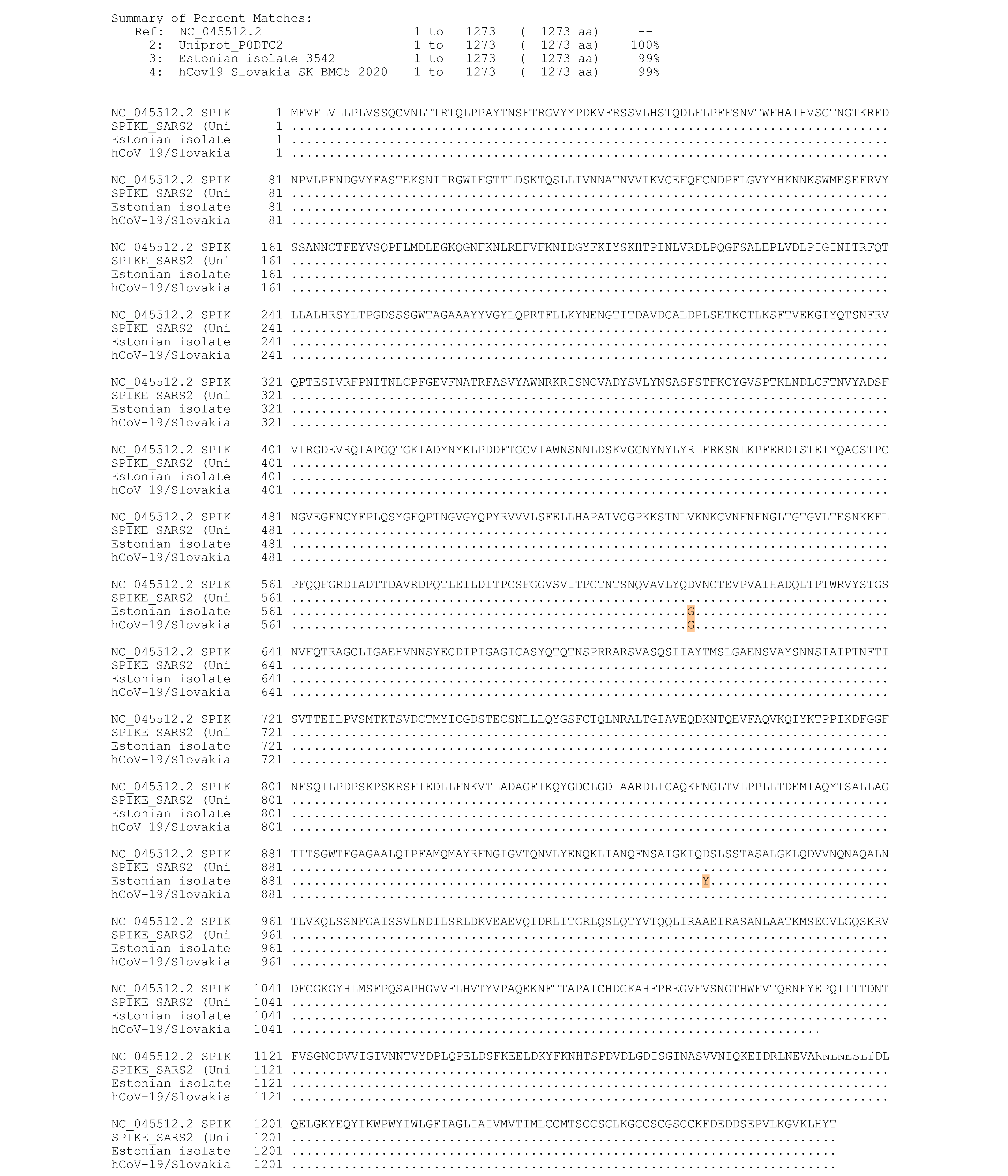

Supplement: S13 Fig — All mutations are highlighted against reference genome extracted from NCBI database: NC_045512.2. (TIFF) [file ppat.1011532.s013.tiff]
