# Supplementary material for: Broadly neutralizing humanized SARS-CoV-2 antibody binds to a conserved epitope on Spike and provides antiviral protection through inhalation-based delivery in non-human primates
Source: PLoS Pathog. 2023 Aug 2;19(8):e1011532. doi: 10.1371/journal.ppat.1011532 (PMC10395824; doi:10.1371/journal.ppat.1011532)
Supplement: S1 Table — (PDF) [file ppat.1011532.s014.pdf]

S1 Table. Cryo-EM data collection, refinement, and validation statistics

	S-trimer + 3 Fab ICO-hu23 (EMDB: EMD-28228)
**Data collection and processing**	
Magnification	105,000
Voltage (kV)	300
Electron exposure (e^-/Å^2)	80
Defocus range (μm)	-1.25 to -2.50
Pixel size (Å)	0.855
Symmetry imposed	C3
Final particle images (no.)	35,845
Map resolution (Å)	3.3
FSC threshold	0.143
Map sharpening B factor (Å^2)	-66
**Refinement**	
Initial model used	6VXX
Model resolution (Å)	3.7
FSC threshold	0.5
Model composition	
Non-hydrogen atoms	29469
Protein residues	3690
Ligands	54
R.m.s. deviations	
Bond length (Å^2)	0.01
Bond angle (°)	1.752
**Validation**	
MolProbity score	1.90
Clashscore	10.75
Rotamer outliers (%)	1.03
Ramachandran plot	
Favored (%)	95.12
Allowed (%)	4.79
Disallowed (%)	0.08
