# Supplementary material for: Broadly neutralizing humanized SARS-CoV-2 antibody binds to a conserved epitope on Spike and provides antiviral protection through inhalation-based delivery in non-human primates
Source: PLoS Pathog. 2023 Aug 2;19(8):e1011532. doi: 10.1371/journal.ppat.1011532 (PMC10395824; doi:10.1371/journal.ppat.1011532)
Supplement: S2 Table — (PDF) [file ppat.1011532.s015.pdf]

S2 Table. Crystallography data collection and refinement statistics.

Sample Name (PDB: ID)	SARS-CoV-2 RBD: Fab ICO-hu23 complex (PDB: 8EL2)
**Data Collection**	
Number of Crystals	1
Diffraction source	APS beamline 23-ID-D
Wavelength (Å)	1.03348
Temperature (K)	100
Detector	Pilatus3 6M
Collection Method	Helical Vector
Crystal-detector distance (mm)	350
Total rotation range (°)	180
Rotation range per image (°)	0.5
Exposure time per image (s)	0.5
Space group	$P3_221$
a, *b*, *c* (Å)	140.30, 140.30, 202.75
α, β, γ (°)	90, 90, 120
Average Mosaicity (°)	0.12
Resolution range (Å)	46.78 – 2.89 (2.98 – 2.89)
Total No. of reflections	521753 (44289)
No. of unique reflections	52265 (4458)
Completeness (%)	99.9 (98.6)
Redundancy	10.0 (9.9)
$\langle I/\sigma(I)\rangle$	11.7 (1.0)
$CC_{1/2}$	0.998 (0.317)
Overall *B* factor from Wilson plot (Å^2)	78.8
**Refinement Statistics**	
Resolution range (Å)	45.92 – 2.89 (2.99 – 2.89)
Completeness (%)	99.7 (97.6)
No. of reflections, working set	52133 (5063)
No. of reflections, test set	2011 (194)
R_{work} / R_{free}	0.239 / 0.282
No. of non-H atoms	9548
Protein	9506
Ligand	41
R.m.s. deviations	
Bonds (Å)	0.006
Angles (°)	1.27
Average *B* factors (Å^2)	99.9
Macromolecules	99.7
Ligand	136.7
Ramachandran plot	
Most favoured (%)	93.78
Allowed (%)	5.32
Outlier (%)	0.90

$CC_{1/2}$ > 0.3 was used to determine the cut-off.
Values for the outer shell are given in parentheses.
