# Supplementary material for: Broadly neutralizing humanized SARS-CoV-2 antibody binds to a conserved epitope on Spike and provides antiviral protection through inhalation-based delivery in non-human primates
Source: PLoS Pathog. 2023 Aug 2;19(8):e1011532. doi: 10.1371/journal.ppat.1011532 (PMC10395824; doi:10.1371/journal.ppat.1011532)
Supplement: S3 Table — The interface interactions are characterized as van der Waals (vdW, 4.0 Å cut-off) or hydrogen bonds (HB, 3.8 Å cut-off). (PDF) [file ppat.1011532.s016.pdf]

S3 Table. Interfacial residues between RBD and Fab ICO-hu23 identified by PISA [42]. The interface interactions are characterized as van der Waals (vdW, 4.0 Å cut-off) or hydrogen bonds (HB, 3.8 Å cut-off).

RBD	Residue	Interaction	Fab ICO-hu23 (H: Heavy chain residues, L: light chain residues,)
444	Lys^{NZ}	HB	H: $Ser75^{O}$
449	Tyr	vdW	H: Tyr27, Thr28, Phe29, Thr30, Thr74, Ser77
	Tyr^{OH}	HB	H: $Tyr27^{O}$, $Phe29^{N}$
455	Leu	vdW	H: Tyr102
456	Phe	vdW	H: Tyr102
484	Glu	vdW	H: Tyr33, Trp50, Asn52, Ser55, Gly57
	Glu^{OE1}	HB	H: $Ser55^{OG}$
	Glu^{OE2}	HB	H: $Tyr33^{OH}$, $Asn52^{ND2}$
485	Gly	vdW	H: Trp104<br>L: Ser97
486	Phe	vdW	H: Trp104<br>L: Tyr32, Tyr34, Thr94, Tyr93, Ser97
489	Tyr	vdW	H: Asn103, Tyr102, Trp104
490	Phe	vdW	H: Ser55, Asn52
492	Leu	vdW	H: Ile54
493	Gln	vdW	H: Thr30, Gly31, Ile54, Tyr102
	Gln^{NE2}	HB	H: $Thr30^{O}$
494	Ser	vdW	H: Ile54
